# DNA sequence-selective G-A cross-linking ADC payloads for use in solid tumour therapies

**DOI:** 10.1038/s42003-022-03633-0

**Published:** 2022-07-29

**Authors:** George Procopiou, Paul J. M. Jackson, Daniella di Mascio, Jennifer L. Auer, Chris Pepper, Khondaker Miraz Rahman, Keith R. Fox, David E. Thurston

**Affiliations:** 1 Femtogenix, Lawes Open Innovation Hub, Rothamsted Research, West Common, Harpenden, Hertfordshire AL5 2JQ UK; 2 School of Biological Sciences, Life Sciences Building B85, University of Southampton, Southampton, Hampshire SO17 1BJ UK; 3 Brighton and Sussex Medical School, University of Sussex, Brighton, BN1 9PX UK; 4 School of Cancer & Pharmaceutical Sciences, Faculty of Life Sciences & Medicine, King’s College London, Franklin-Wilkins Building, 150 Stamford Street, London, SE1 9NH UK

**Keywords:** Cancer immunotherapy, Biochemistry, Drug discovery, Cancer therapy, Computational chemistry

## Abstract

Antibody-Drug Conjugates (ADCs) are growing in importance for the treatment of both solid and haematological malignancies. There is a demand for new payloads with novel mechanisms of action that may offer enhanced therapeutic efficacy, especially in patients who develop resistance. We report here a class of Cyclopropabenzindole-Pyridinobenzodiazepine (CBI-PDD) DNA cross-linking payloads that simultaneously alkylate guanine (G) and adenine (A) bases in the DNA minor groove with a defined sequence selectivity. The lead payload, FGX8-46 (**6**), produces sequence-selective G-A cross-links and affords cytotoxicity in the low picomolar region across a panel of 11 human tumour cell lines. When conjugated to the antibody cetuximab at an average Drug-Antibody Ratio (DAR) of 2, an ADC is produced with significant antitumour activity at 1 mg/kg in a target-relevant human tumour xenograft mouse model with an unexpectedly high tolerability (i.e., no weight loss observed at doses as high as 45 mg/kg i.v., single dose).

## Abstract (web summary)

A class of Cyclopropabenzindole-Pyridinobenzodiazepine (CBI-PDD) DNA cross-linking payloads, used in Antibody-Drug Conjugates, alkylate guanine and adenine bases in the DNA minor groove with a defined sequence selectivity.

## Introduction

An Antibody-Drug Conjugate (ADC) comprises of a monoclonal antibody (mAb) attached *via* a chemical linker to a cytotoxic agent (payload) to provide a targeted therapy for various cancer types^1^. There is currently significant interest in ADCs due to the approval of seven agents by the FDA, polatuzumab vedotin-piiq (Polivy^®^), enfortumab vedotin-ejfv (Padcev^®^), trastuzumab deruxtecan-nxki (Enhertu^®^), sacituzumab govitecan-hziy (Trodelvy^®^), belantamab mafadotin-blmf (Blenrep^®^), loncastuximab tesirine-lpyl (Zynlonta^®^) and tisotumab vedotin-tftv (Tivdak^®^), all between 2019–2022^2^. Furthermore, there are currently over 100 ADCs in clinical trials, with further approvals anticipated in the near future^3^.

**Fig. 1:**
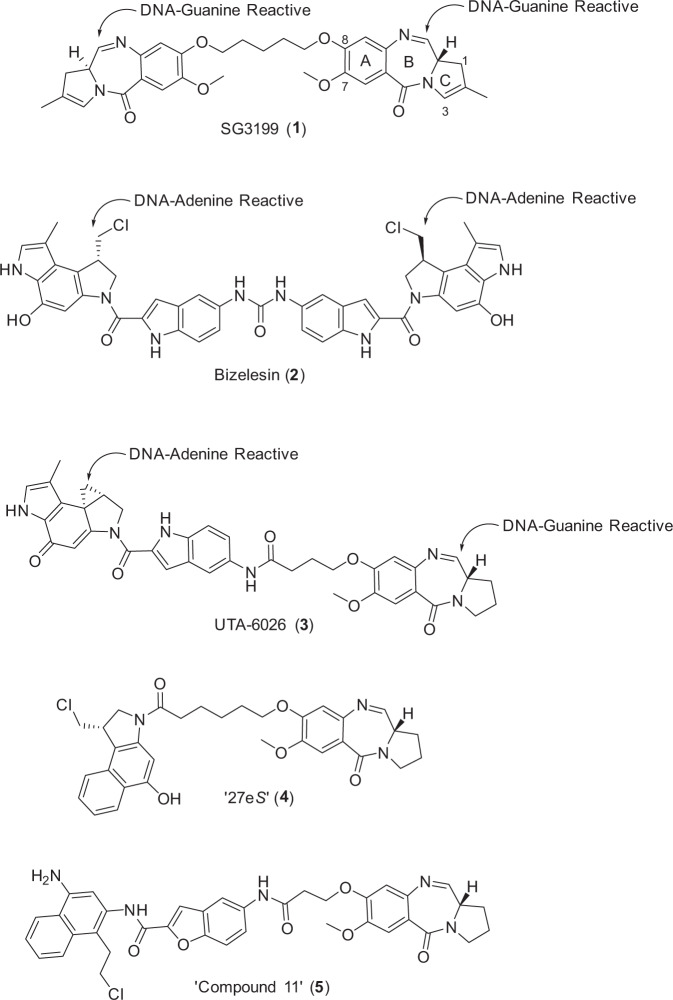
Structures of previously published G-G and G-A DNA cross-linking agents. (**1**) The DNA G-G cross-linking PBD dimer SG3199, the ADC-payload component of Tesirine. (**2**) The A–A cross-linking agent Bizelesin, a cyclopropapyrroloindole (CPI) dimer shown in its *bis*-chloromethyl *seco* (prodrug) form. The three published families of CXI-PBD dimers capable of cross-linking Guanine and Adenine DNA bases: The Hurley et al. (**3**)^11^, Tercel et al. (**4**)^12^ and Lee et al. (**5**)^13^ hybrid dimers.

DNA alkylating and cross-linking agents represent major classes of standalone cancer therapeutics, and examples of compounds of this type have also been developed as ADC payloads. For example, an adenine-alkylating duocarmycin analogue (i.e., *seco*-DUBA) has been used as a payload in Byondis’ trastuzumab duocarmazine (SYD985)^4^, and a guanine-alkylating pyrrolobenzodiazepine (PBD) analogue (i.e., DGN462, indolino-benzodiazepine, IGN) in Immunogen’s IMGN632 (Pivekimab Sunirine)^5^, both of which are in late-stage clinical trials. Most recently, ADCT’s loncastuximab tesirine (Zynlonta^®^), which employs a G-G cross-linking payload (i.e., PBD dimer, SG3199, **1**, Fig. 1), was approved by the FDA in 2021 for use in adult patients with relapsed or refractory B-cell lymphoma^6^. SG3199 (**1**) was also used as a component of StemCentrx’s rovalpituzumab tesirine, and a related PBD dimer payload (SGD-1882) was employed in Seagen’s vadastuximab talirine^7^. However, both of these ADCs failed in the clinic due to modest clinical activity, with investigators concluding that the observed toxicities were payload-related^8^.

Synthetic ‘CXI’ dimers (consisting of two duocarmycin units) capable of cross-linking two adenine bases have also been developed as potential payloads^9^ but no ADCs containing these have yet reached the clinic, potentially due to unfavourable toxicity profiles given that the synthetic dimer bizelesin (**2**, Fig. 1), which forms interstrand A-A cross-links in the DNA minor groove, was evaluated as a standalone therapeutic agent in a Phase I clinical trial but was found to be highly toxic at very low doses with little clinical activity^10^.

Overall, of the approved or clinically-evaluated ADCs described above, for those containing DNA-mono-alkylating or cross-linking agents, all have either failed or only worked effectively in haematological cancers with the exception of trastuzumab duocarmazine (SYD-985) developed by Byondis. This ADC has produced impressive results in the Phase III TULIP® study in patients with pre-treated HER2-positive unresectable locally advanced or metastatic breast cancer (MBC).

Given that DNA-interactive ADC payloads working through G- or A-mono-alkylating or G-G cross-linking mechanisms have reached the clinic, whereas no examples of ADCs with A-A cross-linking payloads have yet progressed to the clinic, we decided to investigate payloads capable of forming G-A cross-links. Non-symmetrical heterodimeric molecules of this type have been previously synthesised by Hurley et al. (i.e., UTA-6026, **3**, Fig. 1)^11^, Tercel et al. (i.e., ′27e*S*′, **4**)^12^ and Lee et al. (i.e., ‘Compound 11’, **5**)^13^, and a Molecular Dynamics Simulation (MDS) study to evaluate the sequence selectivity of these molecules has been reported^14^. An ADC version of **4** has been described and reported to have antitumour activity in xenograft models, although no ADCs containing payloads of this type have been progressed to date^15^.

## Results and discussion

### Molecular modelling and payload design

**Fig. 2:**
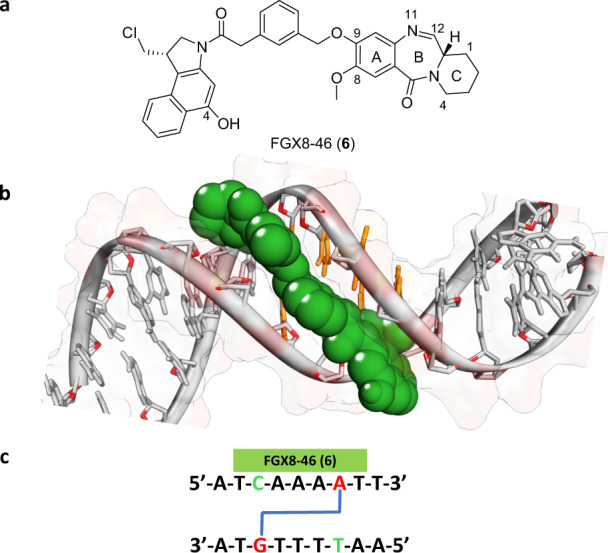
Structure and DNA interaction of the G-A cross-linking ADC payload FGX8-46. **a** Structure of the G-A cross-linking ADC payload FGX8-46 (**6**). **b** Low energy snapshot of a Molecular Dynamics Simulation of **6** binding in the DNA minor groove, with the molecule spanning the seven base-pair sequence 5′-T-C(G)AAAA-T-3′. **c** Details of the sequence spanned in the molecular model (covalently-bound bases in red).

We recently reported a class of DNA G-mono-alkylating ADC payloads, the pyridinobenzodiazepine (PDD) monomers^16,17^. The core unit of a PDD (*cf*. Fig. 2a) has a similar ABC-fused tricyclic system to the PBDs (*cf*. Fig. 1) but the C-ring is expanded to contain six carbons.

Our modelling studies predicted that molecules containing a PDD core should have superior DNA-binding properties compared with PBD-based molecules due to greater flexibility of the six-membered C-ring, thus allowing a more isohelical fit in the DNA minor groove^18^. In support of this, a higher DNA-binding affinity for PDD molecules of this type has been demonstrated by Markandeya and co-workers^19^ using DNA thermal denaturation methodology based on calf thymus DNA. These researchers compared the PBD natural product DC-81, which has a five-membered C-ring, to the analogous PDD molecule containing a six-membered C-ring. At a molar ratio of drug/DNA 1:5 (pH 7.0, 37 °C), they observed increases in DNA melting compared to control (i.e., the ΔT_M_) of 0.3 and 1.2 °C (with no incubation) and 0.7 and 2.2 °C (after 18 h incubation), respectively. Given the improved properties of PDDs for DNA binding, we next used molecular modelling, including several rounds of optimisation, to design the Cyclopropabenzindole-Pyridinobenzodiazepine (CBI-PDD) G-A cross-linker FGX8-46 (**6**) (Fig. 2a) which contains a *meta*-di-substituted benzylic linker. Molecular features studied in the rounds of optimisation included assessment of linker length between the CBI and PDD units, the type of linker (e.g., aryl or alkyl), and orientation of the CBI and PDD units with respect to one another to ensure that the A- and G-alkylating components aligned with relevant DNA bases. Overall, the modelling suggested that **6** should fit isohelically in the DNA minor groove, spanning approximately seven base pairs (Fig. 2b), and with the ability to form interstrand cross-links at 5′-X-C(G)AAAA-X-3′ sequences (covalently-bound bases underlined; X = any base), although the formation of G-A intrastrand cross-links and/or G- or A- mono-alkylated adducts at some sequences could not be excluded.

FGX8-46 (**6**) (Fig. 2a) was deliberately designed to be compatible with most linker technologies. It has two possible linker attachment sites, either the C4-hydroxyl group of the CBI moiety, or the N11-atom of the PDD unit, as utilised in the construction of the linker-payload used in this study (*vide infra*). The C4-hydroxyl group of the CBI unit can also be protected with a chemical grouping to slow or inhibit the A-alkylation event thus creating a prodrug effect, for example using a 4-methylpiperazine-1-carbamoyl moiety^20^ or a phosphate group^21^.

### Synthesis and characterisation of FGX8-46 (6)

FGX8-46 (**6**) was synthesised in 15 steps and 0.98% overall yield (LLS), and purified using standard chromatographic techniques. It was fully characterised by ^1^H- and ^13^C-NMR, LC-MS and HRMS, and determined to be >95% pure by analytical UPLC (see Supplementary Figs. S1-S5, and Supplementary Results S6).

### In vitro cytotoxicity and MTD

FGX8-46 (**6**) was evaluated for cytotoxicity across 11 human cancer cell lines and found to be highly potent, with IC_50_ values ranging from 1.1 picomolar in Reh lymphoblasts to 2.72 nanomolar in the MCF-7 breast cancer cell line, with a median IC_50_ of 0.069 nM across the panel (Table 1).

**Table 1:** IC_50_ values for FGX8-46 (**6**) across a panel of 11 human cancer cell lines after 72 h incubation using an MTS assay (see Methods section).

Cell Line	Cancer Type	IC_50_ (nM)	SD
SK-BR-3	Breast	0.055	0.006
MCF-7	Breast	2.72	0.484
ZR 75-1	Breast	0.486	0.080
U138-MG	Brain	0.434	0.076
Reh	Lymphoblast	0.0011	0.0001
SW48	Colon	0.022	0.003
SW620	Colon	0.069	0.007
LIM1215	Colon	1.30	0.400
RAJI, B	Lymphoblast	0.073	0.024
JVM2	Lymphoblast	0.022	0.003
JURKAT	Lymphocyte	0.047	0.012

The results represent an average value of three determinations for each cell line (Standard Deviations provided in right-hand column).

Overall, **6** was highly cytotoxic in cell lines representing both solid and haematological cancers. For comparative purposes, SG3199 (**1**, Fig. 1) was screened in the SW48, SW620 and LIM1215 cell lines under identical conditions, and produced IC_50_ values of 0.033, 0.92 and 0.30 nM, respectively. This similar level of cytotoxic potency for the two molecules may reflect their DNA cross-linking mechanism of action, albeit for different types of cross-links (i.e., G-G *versus* G-A). **6** was also evaluated for systemic toxicity in a mouse model and found to have a single dose Maximum Tolerated Dose (MTD) of >2.0 mg/kg i.v. (endpoint not reached). This compares favourably with the Tercel G-A cross-linker (**4**, Fig. 1) which was reported to have an MTD of 0.103 mg/kg in a similar mouse model^12^.

### DNA cross-linking assay

**Fig. 3:**
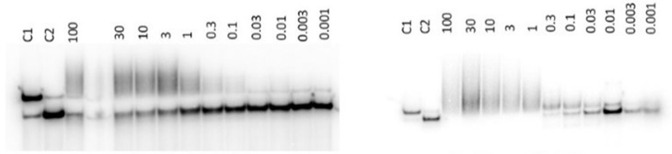
DNA cross-linking assay for the G-A and G-G DNA cross-linking agents FGX8-46 (**6**) (Left Panel) and SG3199 (**1**) (Right Panel). The MS1 DNA sequence^40^ was incubated with 0.001 to 100 *μ*M of either FGX8-46 (**6**) or SG3199 (**1**) in 10 mM Tris-HCl, 0.1 mM EDTA, at pH 7.5 overnight (37 °C). Tracks labelled C1 are dsDNA control lanes in which DNA was not incubated with **6** or **1**. Tracks labelled C2 are control lanes in which DNA was denatured by treating with formamide and then heated to 65 °C for 5 min in the absence of **6** or **1**.

**6** was investigated for its ability to form interstrand DNA cross-links using a modification of an assay developed by Hartley et al.^22^. This involved treating ^32^P-labelled double-stranded DNA with **6** at a range of concentrations (i.e., 0.001 to 100 μM) followed by overnight incubation at 37 °C. Samples were then subjected to denaturing conditions (i.e., 65 °C in formamide for 5 min) followed by analysis on a native polyacrylamide gel. If interstrand cross-links form, the two strands of DNA will be covalently linked and have approximately the same mobility in the gel as double-stranded DNA (dsDNA) (Fig. 3, **Left Panel**). As the CBI component of **6** cleaves DNA at high temperatures due to adenine-alkylation^23,24^, mild conditions were used to perform this assay. Cross-linking started to appear at ~0.3 *µ*M and was not complete by 100 *μ*M. For comparative purposes, the G-G cross-linking payload SG3199 (**1**) was evaluated in the same assay under identical conditions (Fig. 3, **Right Panel**). In this case, cross-linking began at the lower concentration of 0.01 *µ*M, and the DNA was fully cross-linked by 1 *µ*M. These results suggest that **6** is a less efficient DNA cross-linking agent compared with **1** which could explain the higher tolerability profile in mouse models compared to **1** (*vide infra*), as although both molecules possess efficient cell-killing ability (i.e., both are equipotent in the SW48, SW620 and LIM1215 cell lines), G-G cross-links are formed at lower concentrations, potentially explaining the greater systemic toxicity of **1** in animal models.

### DNA sequence-selectivity assay

**Fig. 4:**
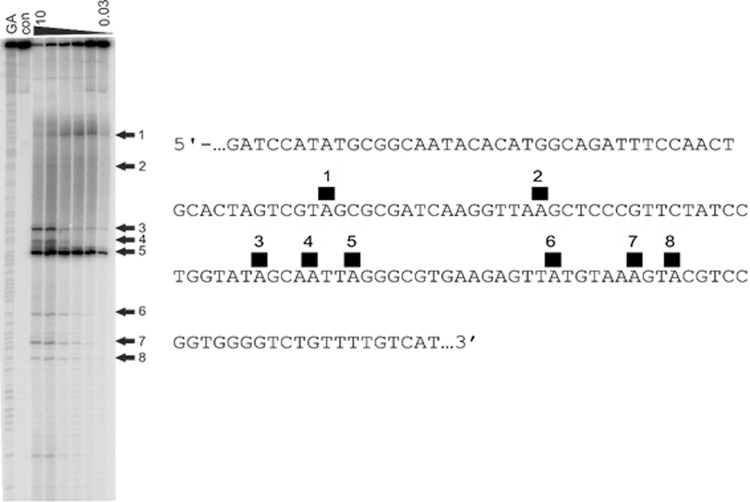
DNA cleavage assay for the G-A cross-linking agent FGX8-46 (**6**) on DNA fragment MS1. The MS1 was incubated with 10, 3, 1, 0.3, 0.1 and 0.03 μM of **6** (indicated at top of gel) in 10 mM Tris-HCl, 10 mM NaCl at pH 7.5 overnight at 37 °C, and a dose-dependent effect is evident (**Left**). The ’GA‘ lane contains Maxam-Gilbert markers specific for purines, and the ‘con’ lane is a control in the absence of **6**. The arrows show the locations of cleavage sites due to the CBI component of **6**, thus indicating covalent A-binding positions. The sequence of the labelled DNA strand is provided (**Right**), with the cleavage sites numbered to correspond to the arrows on the gel.

**Fig. 5:**
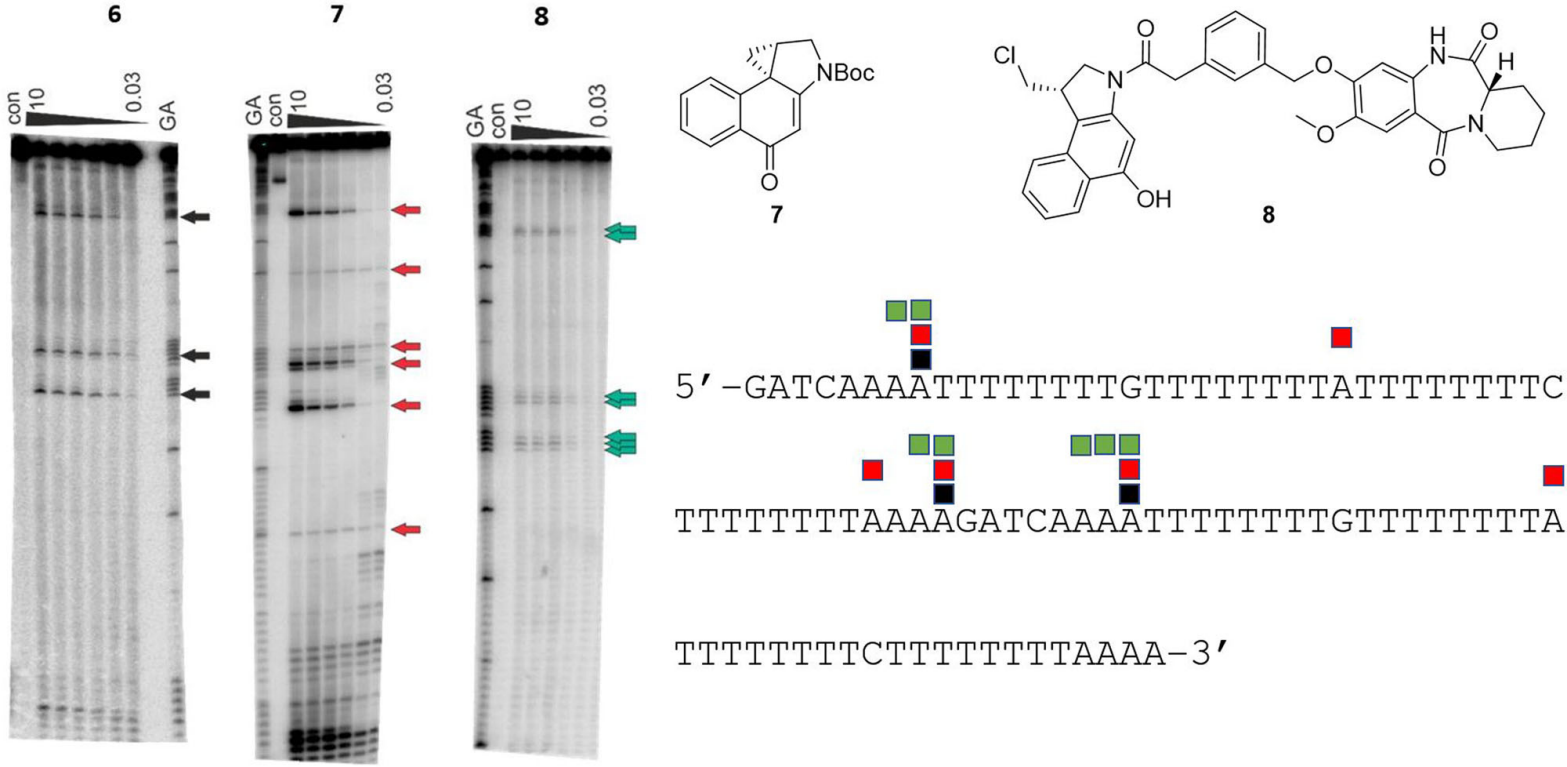
DNA cleavage assay for the G-A cross-linking agent FGX8-46 (**6**), and compounds **7** and **8**, on the 135 base-pair WnGWn DNA fragment that contains isolated guanines within long tracts of adenines and thymines. The WnGWn was incubated with 10, 3, 1, 0.3, 0.1 and 0.3 μM of **6**, **7** and **8** (indicated at tops of gels) in 10 mM Tris-HCl, 10 mM NaCl at pH 7.5 overnight at 37 °C, and a dose-dependent effect is evident (Left). The ‘GA’ lanes contain Maxam-Gilbert markers specific for purines, and the ‘con’ lanes are controls in the absence of **6**, **7** and **8**. The black, red and green arrows on the gels show the locations of cleavage points due to the CBI component of the compounds, thus indicating covalent binding sites. These cleavage sites are also indicated on the DNA sequence using coloured blocks (Bottom Right).

The DNA sequence-selectivity of **6** was evaluated using an assay based on the principle that alkylated adenine bases undergo cleavage upon heating up to 100 °C^23,24^. Using ^32^P-labelled DNA fragments, the precise cleavage sites can be observed on an electrophoresis gel, although for molecules of type **6** this will not allow discrimination between intra- or interstrand cross-linked adducts or mono-adenine-alkylated sites. However, the presence of these different adduct types can sometimes be inferred based on sequence context^23^. Two fragments of DNA were used for this assay, MS1 (Fig. 4) which contains every tetranucleotide combination and WnGWn (Fig. 5) designed to contain more potential cross-linking sites for **6**.

Given the length of the MS1 and WnGWn DNA fragments (i.e., ~200 and 135 base pairs, respectively), the gels shown in Figs. 4 and 5 are surprising in that they contain relatively few cleavage sites suggesting that **6** has a high degree of sequence selectivity, although the formation of mono-G-alkylated adducts cannot be ruled out. However, as the cross-linking gel shown in Fig. 3 (**Left Panel**) provides evidence that **6** can form interstrand cross-links, it is reasonable to assume that adducts of this type represent at least some of the cleavage sites observed in Figs. 4 and 5, with the others potentially due to G-A intrastrand cross-links and/or mono-A-alkylated adducts. According to our molecular model (Fig. 2b), **6** should span approximately seven base pairs, covalently binding to guanine and adenine bases separated by three base pairs. Five of the eight labelled cleavage sites on the Fig. 4 gel (i.e., Sites 1, 2, 5, 7 and 8) are consistent with 5′-AXXXC interstrand cross-linked adducts, and four with intrastrand cross-linked adducts: Sites 2, 3 and 7 (5′-GXXXA) and Site 4 (5′-AXXXG). Site 6 does not contain guanine and adenine bases three base-pairs apart, and so could represent a mono-alkylated A-adduct. Furthermore, in the gel for WnGWn shown in Fig. 5, of the three cleavage sites observed for **6**, the first (starting from the 5′-end) is a mono-A-alkylation site, and the second and third are both consistent with interstrand cross-links (i.e., 5′-AXXXC and 5′-CXXXA, respectively), again suggesting a preference for interstrand cross-links.

To provide further information on DNA adduct formation, the cleavage pattern for **6** on the WnGWn DNA fragment (Fig. 5, **Left-Hand gel**) was compared to those produced by the control molecules **7** (**Middle Gel**) and **8** (**Right-Hand Gel**). Compound **7** consists of the CBI component of **6** alone which should bind to adenine bases with a higher degree of promiscuity than **6**, as there is no requirement for the molecule to locate less common cross-linking sites. Whereas only three cleavage sites were observed on this sequence for the parent molecule **6** (**Left-Hand gel**), the CBI unit alone (**7**) produced six cleavage sites (**Middle Gel**). Similarly, **8**, an analogue of **6** in which the guanine-reactive N11-C12 imine moiety is replaced by a non-electrophilic (i.e., non-covalent-binding) lactam, produced seven cleavage sites (**Right-Hand Gel**). Taken together, the results shown in Figs. 4 and 5 suggest that, as with the PBD dimers^25^, **6** may be able to produce interstrand and intrastrand cross-links and also mono-adducts, but with a slight preference for interstrand cross-links.

### Transcription factor inhibition

Payload **6** was studied in a transcription factor (TF) activation profiling plate array assay (Signosis Inc., USA) designed to monitor the activation of multiple TFs simultaneously to investigate the effect of DNA adduct formation on TF modulation. This assay utilises a series of biotin-labelled DNA probes based on the consensus sequences of TF DNA-binding sites. Nuclear extracts were prepared from HELA cells previously treated with **6** according to the manufacturer’s protocol, which were then incubated with the probe mix. Individual probes find their corresponding transcription factors and form complexes which are separated from free probes through spin column purification. The TF-bound probes are detached from the complex, denatured and then re-hybridised with complementary TF-related sequences coated within individual wells of a plate. The captured biotinylated probes are then detected with a Streptavidin-HRP conjugate (see Supplementary Figures S7A and S7B for partial and full sets of TF array data, respectively).

Of the 95 TFs analysed in the assay, 58 were down-regulated and 37 up-regulated. Another interesting observation was that, for the down-regulated TFs, the extent of down-regulation was significantly greater than for the up-regulated TFs (by up to 12-fold). Of the most down-regulated TFs, notable examples included members of important cancer pathways and regulators of cell cycle such as NFκB, HIF, E2F1 and NF1, and this may contribute to the significant cytotoxicity of **6** (See Supplementary Fig. S7A).

**Fig. 6:**
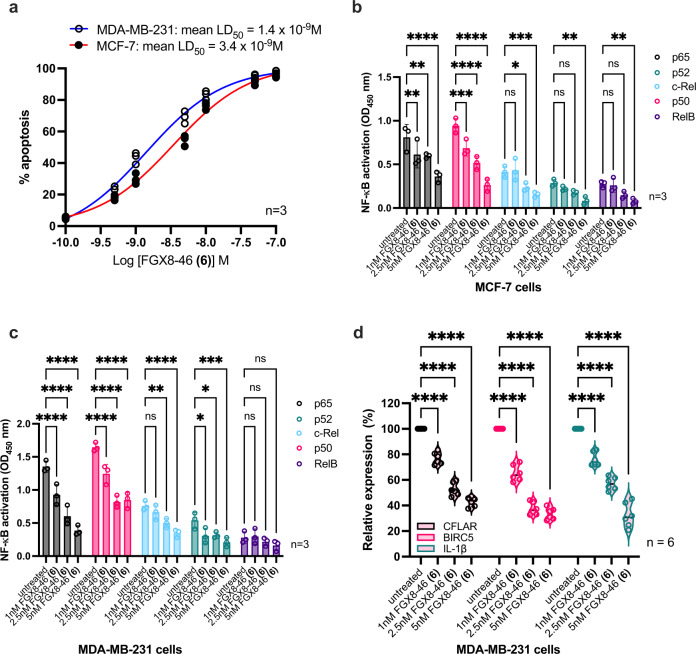
Results of the NF-κB transcription factor studies for the G-A DNA cross-linking agent FGX8-46 (**6**). **a** Determination of LD_50_ values for FGX8-46 (**6**) in MCF-7 and MDA-MB-231 cell lines using Annexin V/7-AAD labelling methodology (*n* = 3); **b**, **c** NF-κB nuclear activity assays in MCF-7 and MDA-MB-231 cell lines, respectively, measuring the effect of 4 h exposure to **6** at concentrations of 1.0, 2.5 and 5.0 nM on P65, p52, c-Rel, p50 and RelB compared to untreated controls (*n* = 3); **d** QRT-PCR validation assays on three NF-κB target genes (CFLAR, BIRC5, IL-1β) in the MDA-MB-231 cell line. Each target transcript was quantitatively assessed using real-time RT-PCR, and the results were normalised to the amount of RPS14 transcript in all samples as an internal house-keeping control (*n* = 6).

In order to further validate the transcription factor inhibitory properties of **6**, additional studies were carried out using two breast cancer cell lines, MCF-7 and MDA-MB-231 (Fig. 6 and Supplementary Data 1). The former was chosen because it was the least sensitive (i.e., IC_50_ = 2.72 nM) within the panel of cell lines in Table 1, and the latter because MDA-MB-231 is a triple negative breast cancer cell line known to have aberrant NF-κB activation^26,27^. Using an Annexin V/7-AAD labelling methodology (Fig. 6a), for MCF-7 an LD_50_ of 3.4 nM was established, consistent with the IC_50_ data in Table 1, and for MDA-MB-231 an LD_50_ of 1.4 nM was observed. NF-κB nuclear activity assays were then carried out with the same cell lines (Figs. 6b, c, respectively), measuring the effect of 4 h exposure to **6** on the P65, p52, c-Rel, p50 and RelB NF-κB subunits at concentrations of 1.0, 2.5 and 5.0 nM compared to untreated controls. A clear concentration-dependent effect was observed for each cell line. Finally, QRT-PCR validation assays were carried out on three NF-κB target genes (CFLAR, BIRC5 and IL-1β) in the MDA-MB-231 cell line. The amount of each target transcript was quantitatively assessed using real-time RT-PCR, and normalised to the amount of RPS14 transcript in all samples, which was used as an internal house-keeping control. The data (Fig. 6d) show that all three genes were significantly repressed by **6** in comparison with the RPS14 control.

### Linker-payload synthesis and conjugation

**Fig. 7:**
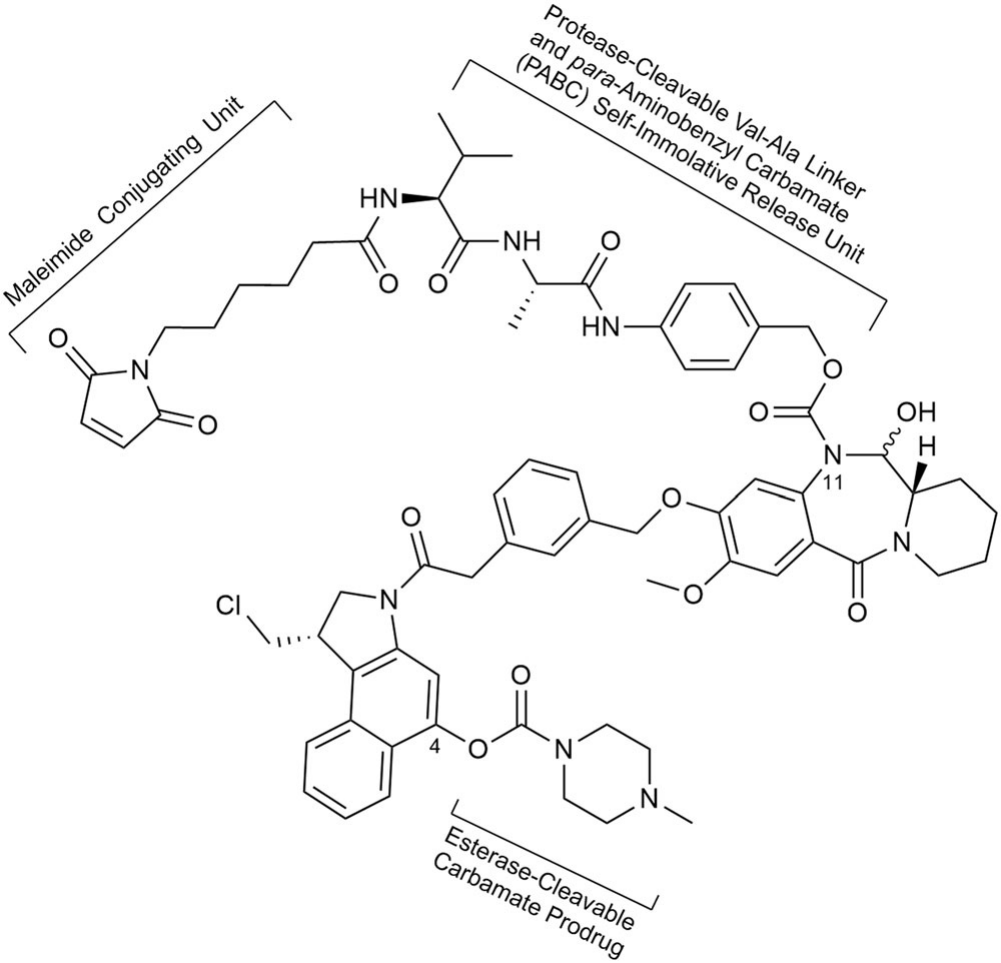
Structure of the Maleimide-Val-Ala-PABC-(FGX8-46) Linker-Payload construct FGX16-11 (**9**).

To demonstrate the suitability of **6** for use as an ADC payload, the linker-payload construct FGX16-11 (**9**, Fig. 7) was synthesised. This construct contains a maleimide moiety (for conjugation to an antibody *via* a thiol group), a cleavable Val-Ala linker with a *para*-aminobenzylcarbamate (PABC) self-immolative release unit at the N11-position of the PDD, and a 4-methylpiperazine-1-carbamate protecting moiety for the C4-hydroxy group of the CBI to avoid its premature activation. There is evidence from the literature^20^ that a 4-methylpiperazine-1-carbamate moiety at this position of a CBI unit will cleave in vivo to release the phenolic group, thus leading to activation of the CBI (i.e., *spiro*-cyclisation) for adenine alkylation. In addition, the electrophilic N11-C12 imine moiety of the PDD unit was converted to a prodrug form by incorporating its N11-position into the *para*-aminobenzylcarbamate (PABC) linkage, thus preventing unwanted reaction with nucleophiles prior to reaching the target cancer cells. In constructs of this type, the PABC functionality has been shown to self-immolate in a cascade initiated by protease-catalysed cleavage of the Val-Ala linker, thus releasing the cytotoxic payload upon internalisation of the ADC in tumour cells^28^. The synthesis of **9** was achieved in 16 steps with 0.66% overall yield (LLS), using a convergent route (see Supplementary Figs. S8–S12 and Supplementary Results S13 for characterisation and analytical data).

The Linker-Payload construct (FGX16-11, **9**) was then conjugated to the EGFR-targeting monoclonal antibody cetuximab with a Drug-Antibody Ratio (DAR) of ~2, using standard stochastic reductive conjugation methodology^29^ to provide the ADC cetuximab-(FGX16-11) (**10**). Due to the hydrophobicity of the maleimide-linker-payload **9**, it was not possible to produce a higher DAR under the conjugation conditions used. However, it should be possible to produce higher DAR species through optimisation of the conjugation conditions, exploration of alternative conjugation strategies, or the addition of water-solubilising chemical groups to the linker and/or payload. The ADC was purified using standard chromatographic methods (i.e., HIC and SEC) and determined to be 96.7% monomer by SEC, the remaining material being dimeric or higher molecular weight species (Supplementary Figs. S14 and S15).

### In vitro cytotoxicity of ADC (10)

The cetuximab-(FGX16-11) ADC (**10**) was not active in in vitro cytotoxicity studies in either the SW-48 (EGFR-positive) or SW-620 (EGFR-negative) cell lines at concentrations up to 10 µM. This was attributed to a’double prodrug effect’, with the relevant enzymes not being available under cell culture conditions to release the 4-methylpiperazine-1-carbamoyl moiety from the C4-position of the CBI unit, or the PABC moiety from the N11-position of the PDD. As the same ADC was active towards SW-48 in the in vivo xenograft model (*vide infra*), this suggests that the relevant enzymes must be present in the mouse and/or the developing tumour tissue.

### In vivo tolerability of ADC (10)

ADC **10** was initially evaluated in a single-dose CD1 mouse study, and it was established that doses as high as 45 mg/kg (i.v.) were tolerated, with concentration-dependent weight loss the only toxicity observed. These doses were unexpectedly high given the generally low tolerability profile of PBD dimer based ADCs. For example, Miller et al. have reported^30^ that anti-FRα ADCs based on either mono-G-alkylating IGN or G-G cross-linking PBD dimers have single-dose MTDs in mice of 6 mg/kg and 2.8 mg/kg, respectively, and a single dose MTD of 1.5 mg/kg in rats has been reported for another ADC employing SG3199 (**1**) as payload^31^. Also, when an ADC version of the Tercel G-A cross-linker 27e*S* (**4**) based on the hu7C2 HER2-targeted antibody was assessed in an antigen-independent rat toxicity model, an MTD of 15 mg/kg was observed. Interestingly, in the same study, an MTD of <5 mg/kg was observed for an equivalent ADC containing an A-A cross-linking CBI dimer payload, along with MTDs of 10 mg/kg and 7.5 mg/kg for equivalent ADCs containing a G-G cross-linking PBD dimer payload with cleavable and non-cleavable linkers, respectively^32,33^. Based on allometric scaling^34^, the rat MTD of 15 mg/kg observed for the Tercel G-A cross-linker is approximately equivalent to an MTD of 30 mg/kg in mice, although the limitations of allometric scaling between species are well-recognised. Thus, the payload **6** (FGX8-46) appears to produce an ADC with an improved tolerability profile compared to those based on either 27e*S* (**4**), which has an identical mechanism of action, or the G-G cross-linking SG3199 (**1**), although the different antigen targets and linker types may also contribute to this.

**Fig. 8:**
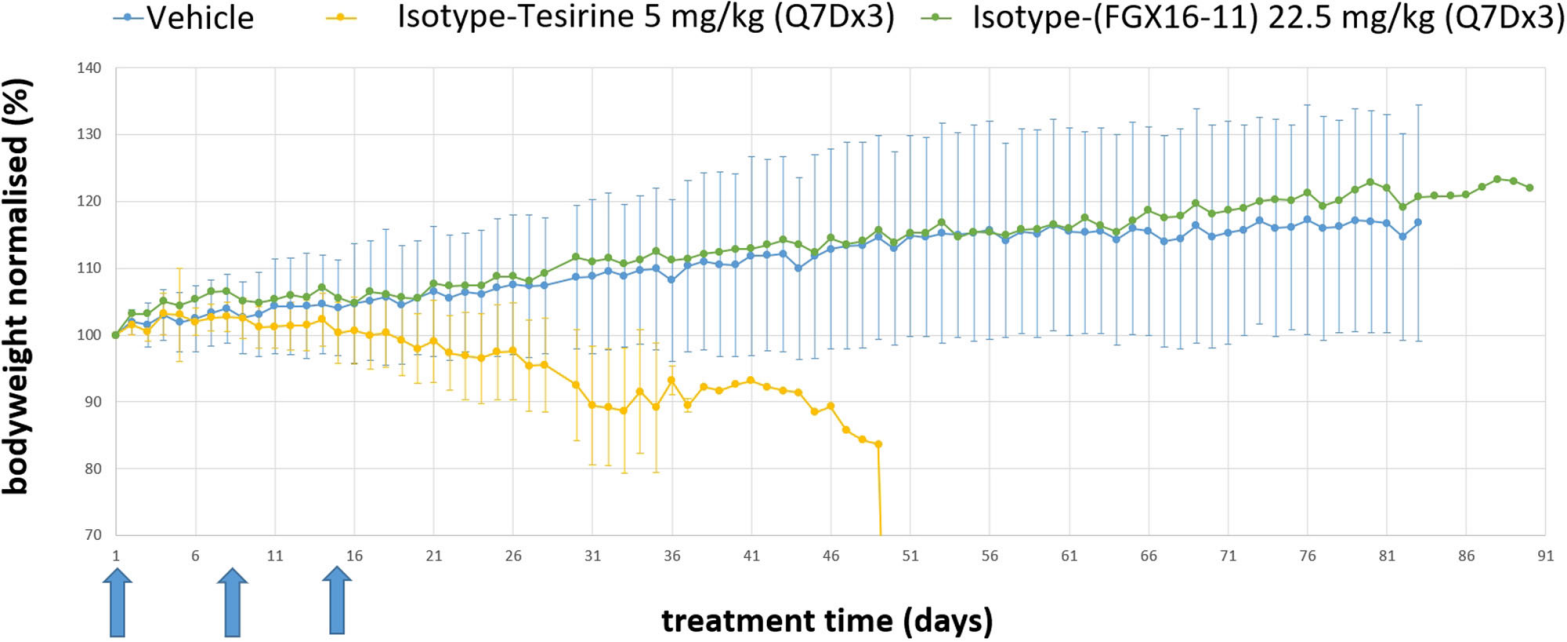
In vivo tolerability study showing the effect of the non-targeting Isotype-(FGX16-11) ADC dosed at 22.5 mg/kg every seven days (Q7Dx3) on the body weight of mice out to 90 days. Data for the equivalent Isotype-tesirine ADC dosed at 5 mg/kg (Q7Dx3) is shown for comparative purposes. The blue arrows represent the Q7Dx3 dosing points (*n* = 6 mice per cohort).

To investigate this further, the potential for delayed toxicity in mice was explored by producing an ADC from a non-targeting (anti-GFP) antibody and **6** (i.e., Isotype-(FGX16-11)), monitoring bodyweight out to 90 days. In this experiment, an equivalent ADC (Isotype-tesirine) containing the G-G cross-linking PBD dimer SG3199 as payload (**1**) was included. As evident from Fig. 8 (See also Supplementary Data 2 and 3), mice in the Isotype-(FGX16-11) cohort dosed at 22.5 mg/kg on days 0, 7 and 14 (i.e., Q7Dx3) showed no sign of toxicity with all animals gradually gaining weight out to 90 days. However, mice in the Isotype-tesirine cohorts dosed at 5 mg/kg on days 0, 7 and 14 (Q7Dx3) showed a downward trend in body weight from the first administration and, by day 50, all mice had to be sacrificed due to weight loss and hind-limb paralysis. It is interesting to note that, although the double prodrug structure of **9** within Isotype-(FGX16-11) may be a contributing factor toward its enhanced tolerability profile relative to Isotype-tesirine, ADCs based on similarly prodrugged PBD dimers still produce cumulative toxicity^35^, suggesting that the G-A cross-linking mechanism of action may be inherently less toxic.

### In vivo efficacy of ADC (10)

**Fig. 9:**
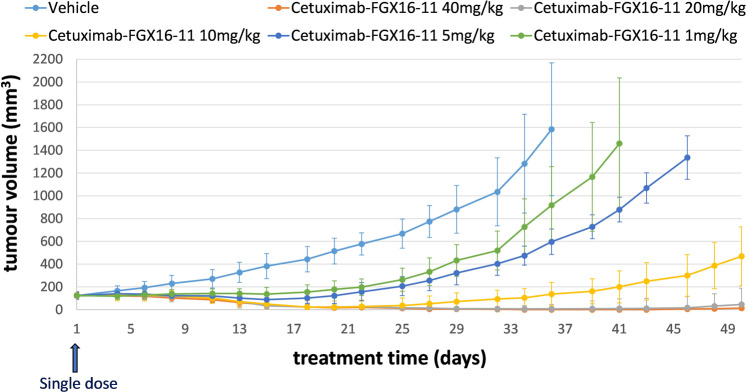
Results of an in vivo efficacy study of Cetuximab-(FGX16-11) (**10**) in a Human Tumour Xenograft experiment based on BALB/c mice and the EGFR-expressing human colon cancer cell line SW-48 at single doses ranging from 1 mg/kg to 40 mg/kg. The ADC was active at all levels including the lowest 1 mg/kg (single dose), which provided tumour suppression out to approximately three weeks; *n* = 6 mice per cohort.

A single-dose in vivo efficacy study was undertaken in a Human Tumour Xenograft model using BALB/c mice and the EGFR-expressing human colon cancer cell line SW48 (Fig. 9 and Supplementary Data 4). ADC (**10**) was administered i.v. (tail vein) to six cohorts of mice (6 mice per cohort) at dose levels of 1, 5, 10, 20 and 40 mg/kg. The ADC was active at all doses, with the lowest dose (i.e., 1 mg/kg) providing tumour suppression out to approximately three weeks. The higher dose of 20 mg/kg provided complete tumour suppression out to 50 days. It is possible that repeat administration at lower dose levels such as 1 mg/kg every three weeks may provide tumour suppression for a longer time period provided tumour resistance does not occur.

As the ADC **10** is tolerated at doses as high as 45 mg/kg in mice, this extrapolates to a tolerated dose of ~5.6 mg/kg in humans *via* allometric scaling (i.e., division by 8 from mouse to human)^34^. If correct, this should allow dosing in the 2.5–5 mg/kg range (Q7D or Q21D) in humans, consistent with the treatment of solid tumours. Two recent reports on the HER2-targeted ADC (DHES0815A), which contains a G-mono-alkylating payload^36,37^, provide additional context on allometric scaling. For DHES0815A dosed every three weeks (x 5) in cynomolgus monkeys, an HNSTD (Highest Non-Severely Toxic Dose) of 12 mg/kg was observed which translated into an MTD of 2.4 mg/kg in humans in the subsequent Phase 1 study (i.e., 12/4.75, rather than 12/2 as estimated by allometric scaling^34^). Although DHES0815A contains a G-mono-alkylating rather than a G-A cross-linking payload, the observed HNSTD in cynos of 12 mg/kg is similar to the estimated 11.25 mg/kg for ADC **10**, thus supporting the potential for **10** to be dosed in the 2.5-5 mg/kg range in humans (i.e., 11.25/4.75 = ~2.4 mg/kg), consistent with the treatment of solid tumours.

## Conclusions

An ADC payload class, the CBI-PDD G-A cross-linkers, has been designed, a member of which (**6**, FGX8-46) has been evaluated as a component of an EGFR-targeted ADC (**10**). Gel-based biophysical studies have established that **6** has a unique DNA sequence-selectivity with a preference for binding to sequences containing adenine and guanine bases separated by three base pairs (e.g., 5′-AXXXC), and with a small preference for interstrand rather than intrastrand cross-linking. Although approximately equivalent in in vitro cytotoxicity to G-G cross-linking PBD dimers such as SG3199 (**1**) in human tumour cells lines, **6** has been shown to produce an efficacious ADC with an improved tolerability profile. Although the reason for this is not fully understood, it may relate to the weaker covalent bond between the CBI unit and the adenine N3-atom compared to the stronger bond between a PBD/PDD unit and the C2-NH_2_ group of a guanine. This is evident from the gel experiments reported here which show that the CBI/adenine-N3 bond can be cleaved at higher temperatures while leaving the PDD/guanine-N2 bond intact. It is conceivable that this may lead to a more facile repair of G-A cross-links in healthy cells compared to G-G cross-links which could explain the greater tolerability. However, this is a complex situation as the different DNA sequence-selectivity of **6** compared with a PBD dimer could directly affect the types and/or rates of relevant repair mechanisms in both healthy and cancer cells. Therefore, further mechanistic studies are required to elucidate the role of these differences on tolerability. Also, the transcription factor studies have shown that **6** can down-regulate a number of key cancer-related transcription factors which may contribute to its activity.

The relative contributions of the C4-carbamate prodrug moiety, the lower cross-linking efficacy, or the GA-sequence-selectivity of **6** to the high tolerability of cetuximab-(FGX16-11) are presently unclear. However, for the discontinued ADC MDX-1203 developed by Medarex which contains a similar mono-A-alkylating CBI-based unit, the C4-prodrug moiety was considered to play an important role, with cleavage of the protecting group postulated to occur in the endoplasmic reticulum by the human enzyme Carboxylesterase 2^38^. If it becomes apparent that cleavage of the C4-carbamate prodrug moiety of **10** provides a significant contribution to its efficacy, then the fact that mice have higher levels of esterases compared to humans may be a risk factor in transitioning from pre-clinical studies to clinical trials.

Overall, the results reported here suggest that **6** could offer advantages over currently available DNA covalent-binding ADC payloads as it is well tolerated when incorporated into an ADC, is not associated with late-onset toxicity, and is capable of being used in a multi-dose schedule. As the cetuximab-(FGX16-11) ADC (**10**) is tolerated in mice at doses of up to 45 mg/kg, this suggests that ADCs based on the G-A cross-linking payload **6** may be better tolerated in humans than those based on G-G cross-linking or mono-G-alkylating payloads. Therefore, this payload type is worthy of further investigation as it could be a useful component of next-generation ADCs, particularly with a view to treating solid tumours and overcoming resistance to ADCs containing other payload types.

## Methods

### Molecular modelling

Molecular models were constructed using the AMBER (v11) software suite with DNA sequences constructed using the *nab* module. The ligand was constructed using Maestro followed by conversion to mol2 files (with the application of Gasteiger charges) using *antechamber*. *Parmchk* was then used to determine missing parameters for the ligand. The gaff and DNA-optimised parm99bsc0 force-fields were loaded for DNA, and *xleap* was used to manually dock the ligand and create the covalent bond between the N11-C12 imine moiety of the PDD and the C2-amino of the reacting guanine, and between the alkyl halide of the CBI and the N3 of an adenine base. Covalent attachment of the PDD was based on the crystallographic structure of anthramycin^39^. An identical process was performed with the PDD of **6** in its N11-C12 imine form and the CBI in its alkyl halide form to understand non-covalent interactions with the DNA sequence. Parameters for covalent attachment of the PDD and CBI to the DNA structure were derived from in-house studies. Each adduct was minimised in a gradient manner by initially placing the DNA under a high force constant to enable the ligand to find its energy minimum, followed by reduction in force and a relaxation of restraints in a periodic manner. Once the full system was minimised, production simulations were run in implicit solvent for a period of 20 ns using *pmemd*. The generalised Born/surface area (GB/SA) implicit solvent model was used, with monovalent electrostatic ion screening simulated with SALTCON set to 0.2 M. The dynamics integration time-step was 0.002 ps while constraining all bonds to hydrogen atoms using the SHAKE algorithm. A temperature of 300 K was maintained using the Langevin thermostat, and a long range non-bonded cut-off of 50 Å was used. Molecular dynamics simulations were analysed using the VMD software, and images were created using the Chimera (Version 1.11) software.

### In vitro cytotoxicity assay

Cell-lines were established and, after 72-hour incubation with **1** or **6**, the cell proliferation of each sample was measured using the Cell Titre 96 AQueous One Solution Cell Proliferation Assay (Promega #G3582) according to the manufacturer’s protocol. The percentage inhibition was calculated against the mean of the control-treated samples. IC_50_ values for inhibition of cell growth were determined with the GraphPad Prism software using non-linear regression (4 parameter logistic equation) with bottom and top constraints at 0 and 100%, respectively. Cells were sourced from ATCC.

### Preparation of ^32^P-labeled DNA fragments MS1 and WnGWn

The DNA sequence MS1^40^ containing every possible tetranucleotide sequence, and WnGWn designed to contain isolated guanines within long tracts of adenines and thymines, were cloned into the BamHI site of pUC18. Plasmids containing the sequences of interest were digested with HindIII and EcoRI, and labelled at the 3’-end of the HindIII site using α-^32^P[dATP] with the Klenow fragment (exo-). The labelled DNA was separated from the remainder of the plasmid on a 6% polyacrylamide gel, and then eluted and dissolved in 10 mM Tris-HCl pH 7.4 containing 0.1 mM EDTA.

### Cross-linking assay

1.5 µL of the cross-linking agent (**1** or **6**) was diluted in 10 mM Tris-HCl pH 7.4 containing 10 mM NaCl, and then added to 1.5 µL of radiolabelled MS1 DNA at concentrations between 100 µM – 1 nM. The complexes were incubated overnight at 37 °C, then denatured by adding 5 µL formamide containing 10 mM EDTA, before heating at 65 °C for 5 min and then placing on ice. Control non-denatured duplex DNA (Control 1) was prepared in the same way, but was not heated to 65 °C. Samples were run on 6.4% native polyacrylamide gels at 100 V in 1 X TBE running buffer. Gels were then fixed in 10% acetic acid for at least 10 min and transferred to Whatman 3 mm paper. After drying under a vacuum at 80 °C, the gels were exposed to a phosphorimager screen overnight and imaged using a Typhoon FLA 7000 phosphorimager.

### DNA cleavage assay

Both the MS1 and WnGWn DNA fragments were used in the cleavage experiments in order to determine the binding sites of **6**, **7** and **8**. Stocks of the compounds were diluted in Tris-HCl pH 7.4 containing 10 mM NaCl, and 1.5 µL of each diluted compound was mixed with 1.5 µL of the radiolabelled DNA and incubated at 37 °C overnight. Heating CBI-DNA complexes results in cleavage at the adenines to which the CBI-components are attached. Therefore, 4.5 µL of formamide containing 10 mM EDTA was added to the ligand-DNA complexes followed by heating at 100 °C for 3 min, before cooling on ice and loading onto an 8% denaturing polyacrylamide gel containing 8 M urea. The gels were fixed in 10% acetic acid for 10 min, transferred to Whatman 3 mm paper and dried under vacuum at 80 °C. They were then exposed to a phosphorimager screen overnight and imaged using a Typhoon FLA 7000 phosphorimager.

### Transcription factor plate array assay

The transcription factor plate array assay kit was obtained from Signosis, Inc (USA). 2 × 10^6^ HeLa cells were treated with 100 nM of **6**, and incubated for 6 h before extracting the nuclear protein and carrying out the TF plate array assay according to the manufacturer’s instructions. In the case of each transcription factor, the RLU value obtained for the cells treated with **6** was deducted from the respective values obtained for the cells treated with vehicle control to obtain the differences in TF activation/inhibition (see Supplementary Figs. S7A and S7B for partial and full sets of TF array data, respectively).

### Annexin V/7-AAD labelling, NF-kB nuclear activity, and QRT-PCR validation assays: cell culture conditions

Cells were sourced from ATCC. MCF-7 and MDA-MB-231 cells were cultured in DMEM and RPMI media, respectively (Invitrogen), both supplemented with 10% foetal calf serum and 100 units/mL penicillin and 100 μg/mL streptomycin. Cells were aliquoted (10^5^ cells in 500 μL of media) into 24-well plates and incubated at 37 °C in a humidified 5% carbon dioxide atmosphere in the presence of **6** (1 × 10^−10^ −1 × 10^−7^ M) for up to 72 h. In addition, control cultures were set-up in which no drug was added. Cells were subsequently harvested by centrifugation, and then analysed by flow cytometry using the methods outlined below. Experiments were performed in either duplicate or triplicate.

### Measurement of in vitro apoptosis

Harvested cells were re-suspended in binding buffer containing 4 μL of annexin V labelled with allophycocyanin (APC) (Biolegend). Cells were subsequently labelled with 7-AAD, and apoptosis was quantified using a CytoFLEX LX flow cytometer (Beckman Coulter). At least 10,000 events were acquired, and data were subsequently analysed using the FlowJo software. LD_50_ values (the concentration of **6** required to kill 50% of cells) were interpolated from the dose-response curves.

### Nuclear NF-κB subunit detection by ELISA

MCF-7 and MDA-MB-231 cells were treated with **6** (0, 1 nM, 2.5 nM, 5 nM) for 4 h. Subsequently, nuclear proteins were isolated using a nuclear extract kit (Active Motif). Extracts were then assayed for NF-κB subunit DNA binding with a TransAM NF-κB family kit according to the manufacturer’s protocol. The consensus oligonucleotide used for NF-κB binding was 5′-GGGACTTTCC-3′, and specific NF-κB proteins bound to the oligonucleotide were identified using primary antibodies for NF-κB subunits p50, p52, p65, c-Rel and RelB, followed by detection with an HRP-conjugated secondary antibody. The absorbance at 450 nm (*A*_450_) was read on a microtitre plate reader (Bio-Rad), and readings were compared with the untreated controls.

### Real-time reverse transcription-PCR

MDA-MB-231 and MCF-7 cells were treated with **6** (0, 1 nM, 2.5 nM, 5 nM) for 4 h. Aliquots of 1 × 10^6^ cells were then harvested by centrifugation prior to RNA extraction using RNeasy mini-spin columns according to the manufacturer’s instructions (Qiagen). RNA (1 μg) was then reverse transcribed in 20 μL reactions containing 10× Buffer II, 5 mmol/L MgCl_2_, 0.5 μmol/L deoxynucleotide triphosphates, 2.5 units reverse transcriptase, 1 unit of RNase inhibitor, and 2.5 μmol/L random hexamers. cDNA (2 μL) was used in each RT-PCR reaction. PowerTrack SYBR Green Mastermix (ThermoFisher Scientific) was used to quantify the amount of transcript present in each sample using primer pairs for the NF-κB target genes: CFLAR, BIRC5 and IL-1β. All primers were purchased from Eurogentec Ltd (Southampton, UK). The amount of target transcript was assessed using real-time RT-PCR using a QuantStudio 7 instrument (ThermoFisher Scientific), and was normalised to the amount of RPS14 transcript in all samples as an internal house-keeping control. The results of the real-time RT-PCR (Fig. 6d) are presented as normalised target gene values (e.g., the ratio between CFLAR and RPS14 transcripts calculated from the crossing points of each gene). All experiments were performed in triplicate. cDNA was amplified using the following primers:

CFLAR: 5′-gtggagacccacctgctca-3′ (forward) and 5′-ggacacatcagatttatccaaatcc-3′ (reverse);

BIRC5: 5′-ttagcagaaaatgcactccag-3′ (forward) and 5′-ctggttttaaggatggccttt-3′ (reverse);

IL-1β: 5′-tggcagaaagggaacagaaa-3′ (forward) and 5′-acttcttgccccctttgaat-3′ (reverse);

RPS14: 5′-ggcagaccgagatgaactct-3′ (forward) and 5′-ccaggtccaggggtcttggt-3′ (reverse).

### Statistics and reproducibility

All statistical analyses were performed using Graphpad Prism 9 software (Graphpad Software, Inc.). Drug sensitivity to **6** was evaluated using non-linear regression and line of best fit dose-response curves. Comparison of relative gene transcription and NF-kB subunit nuclear activity following exposure to **6** for 4 h were assessed using two-way ANOVA with Dunnett’s correction for multiple comparisons. The Flow Cytometry Gating Strategy used is provided in Supplemental Methods S16 and Supplementary Fig. S17.

### Conjugation and ADC purification

The interchain disulfides of commercially-available cetuximab formulated at pH 7-8 in 2 mM EDTA were partially reduced with TCEP for 90 – 180 min. The extent of reduction was controlled to achieve a specified drug-to-antibody ratio (DAR) of ~2. The reduced antibody was then diluted with PBS/2 mM EDTA to 2 mg/mL. FGX16-11 (**9**) was dissolved in DMA/DMSO, and conjugation to the antibody was achieved by addition of an excess of **9** to a 1:1 mixture of the reduced antibody and propylene glycol, at a final protein concentration of 1 mg/mL. The mixture was allowed to react for 1 h to form the antibody-drug conjugate **10**, and the reaction was then quenched by adding an excess of *N*-acetyl cysteine to remove unreacted **9**. The mixture was further diluted with 1:1 PBS/3% cyclodextrin and added to a proprietary purifying resin. The resin-bound ADC was washed with PBS/3% cyclodextrin to remove excess small-molecule impurities, and then released from the resin. The ADC was finally formulated through G25 desalting into PBS/3% cyclodextrin, and filtered prior to aliquoting and storing at −80 °C. HIC and SEC chromatograms for the final ADC product are provided in Supplementary Figs. S14 and S15.

### In vivo tolerability studies

A total of 56 male CD1 mice were used for the tolerability study. All animals were allowed free access to a standard certified commercial diet and sanitised water during the study. The holding room was maintained under standard conditions: 20–24 °C, 40–70% humidity and a 12 h light/dark cycle. All protocols used in this study were approved by the CRO’s Animal Welfare and Ethical Review Committee, and all procedures were carried out under the guidelines of the Animal (Scientific Procedures) Act 1986. Mice were randomly assigned to a treatment group and dosed intravenously through the tail vein with the free payload (**6**) or ADC (**10**). The weight of the mice was monitored for a period of 28 days, and the Maximum Tolerated Dose (MTD) was deemed to have been reached when >15% weight loss of a mouse was observed. The minimum dose at which >15% weight loss was observed was considered to be the MTD.

### Delayed toxicity study

A total of 54 male ICR-CD1 mice aged 5–8 weeks were used for the study. These were purchased from Envigo and allowed to acclimatise for 7 days prior to dosing. Animals were housed in IVC cages (up to 5 per cage) with individual mice identified by tail marks. All animals were allowed free access to a standard certified commercial diet and sanitised water during the study. The holding room was maintained as follows: room temperature at 20–24 °C, humidity at 30–70% and a 12 h light/dark cycle. All protocols used in this study were approved by the CRO’s Animal Welfare and Ethical Review Committee, and all procedures were carried out under the guidelines of the Animal (Scientific Procedures) Act 1986. The mice were randomly assigned to treatment groups, and animals received a dose of each compound either Q7D or Q21D (data not shown) for 3 cycles, with bodyweight and health observations made daily for up to 90 days. The ADCs were prepared for injection in phosphate buffered saline (PBS) pH = 7.4 with 3% w/w cyclodextrin. Dosing solutions were prepared from stock solutions immediately prior to dosing, and any remaining dosing solutions were discarded.

### Efficacy study

Cells were sourced from ATCC. A total of 36 male BALB/c nude mice aged 6–8 weeks was used for the efficacy study. SW48 cells (1 × 10^7^, 1:1 with Matrigel) were implanted into the flank of a mouse using a 23-gauge needle. Once tumours reached 150 mm^3^, the mice were randomly assigned to the relevant treatment groups and then dosed intravenously through the tail vein with ADC **10**. Tumour volume was measured daily using standard methodology based on digital callipers. The length and width of the tumour was measured and the volume calculated using the following formula: volume = (length × width^2^)/2, where length represents the largest tumour diameter and width represents the perpendicular tumour diameter.

### Reporting summary

Further information on research design is available in the Nature Research Reporting Summary linked to this article.

## Supplementary information

Supplementary Information

Description of Additional Supplementary Files

Supplementary Data 1

Supplementary Data 2

Supplementary Data 3

Supplementary Data 4

Reporting Summary

## Data Availability

Key data supporting the findings of this study are available within this article, the corresponding Supplementary Information document, and the Supplementary Data 1 to 4 files.
