# Supplementary material for: DNA sequence-selective G-A cross-linking ADC payloads for use in solid tumour therapies
Source: Commun Biol. 2022 Jul 29;5:741. doi: 10.1038/s42003-022-03633-0 (PMC9338023; doi:10.1038/s42003-022-03633-0)

DNA Sequence-Selective G-A Cross-Linking ADC Payloads for Use in Solid Tumour Therapies

George Procopiou,[a] Paul J. M. Jackson,[a] Daniella di Mascio,[b] Jennifer L. Auer,[a] Chris Pepper,[c] Khondaker Miraz Rahman,[a][d] Keith R. Fox[b] and David E. Thurston*[a][d]

[a] Femtogenix, Lawes Open Innovation Hub, Rothamsted Research, West Common, Harpenden, Hertfordshire, AL5 2JQ, UK.

[b] School of Biological Sciences, Life Sciences Building B85, University of Southampton, Southampton, Hampshire, SO17 1BJ, UK.

[c] Brighton and Sussex Medical School, University of Sussex, Brighton, BN1 9PX, UK.

[d] School of Cancer & Pharmaceutical Sciences, Faculty of Life Sciences & Medicine, King's College London, Franklin-Wilkins Building, 150 Stamford Street, London, SE1 9NH, UK.

* Corresponding author: david.thurston@femtogenix.com

Table of Contents **Page**

Supplementary Figure S1: ^{1}H NMR of FGX8-46 (**6**)	3
Supplementary Figure S2: ^{13}C NMR of FGX8-46 (**6**)	4
Supplementary Figure S3: LCMS of FGX8-46 (**6**)	5
Supplementary Figure S4: Analytical HPLC of FGX8-46 (**6**)	6
Supplementary Figure S5: HRMS of FGX8-46 (**6**)	7
Supplementary Results S6: Characterisation Data for FGX8-46 (**6**)	8
Supplementary Figures S7A and S7B: Partial and Full TF Array Data Sets	9 and 10
Supplementary Figure S8: ^{1}H NMR of FGX16-11 (**9**)	11
Supplementary Figure S9: ^{13}C NMR of FGX16-11 (**9**)	12
Supplementary Figure S10: LCMS of FGX16-11 (**9**)	13
Supplementary Figure S11: Analytical HPLC of FGX16-11 (**9**)	14
Supplementary Figure S12: HRMS of FGX16-11 (**9**)	15
Supplementary Results S13: Characterisation Data for FGX16-11 (**9**)	16
Supplementary Figure S14: HIC Chromatogram of Cetuximab-(FGX16-11) (**10**)	17
Supplementary Figure S15: SEC chromatogram of Cetuximab-(FGX16-11) (**10**)	17
Supplementary Methods S16: Flow Cytometry Gating Strategy	17
Supplementary Figure S17: Gating Strategy	18

Supplementary Figure S1: ^{1}H NMR of FGX8-46 (6)

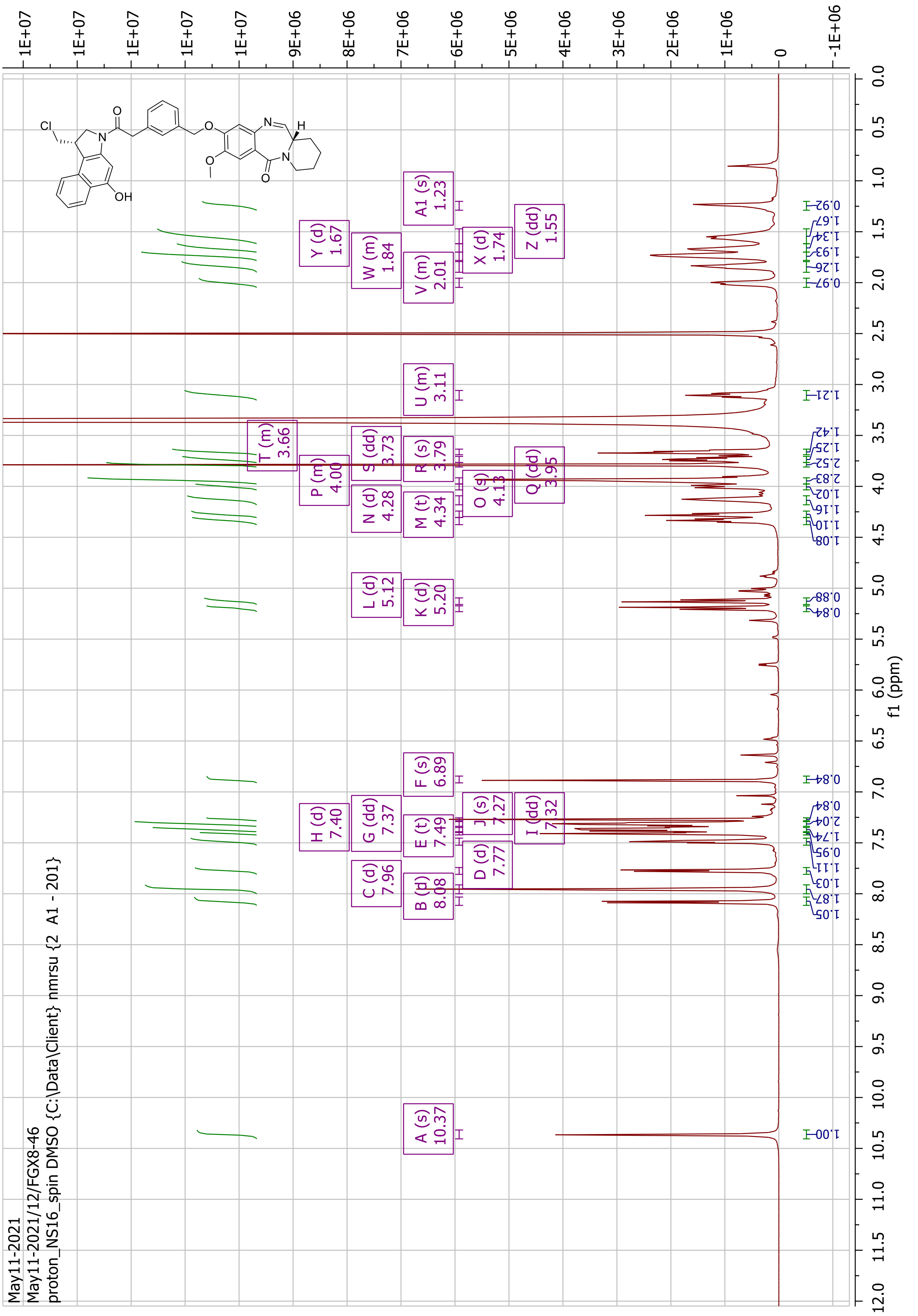

Supplementary Figure S2: ^{13}C NMR of FGX8-46 (6)

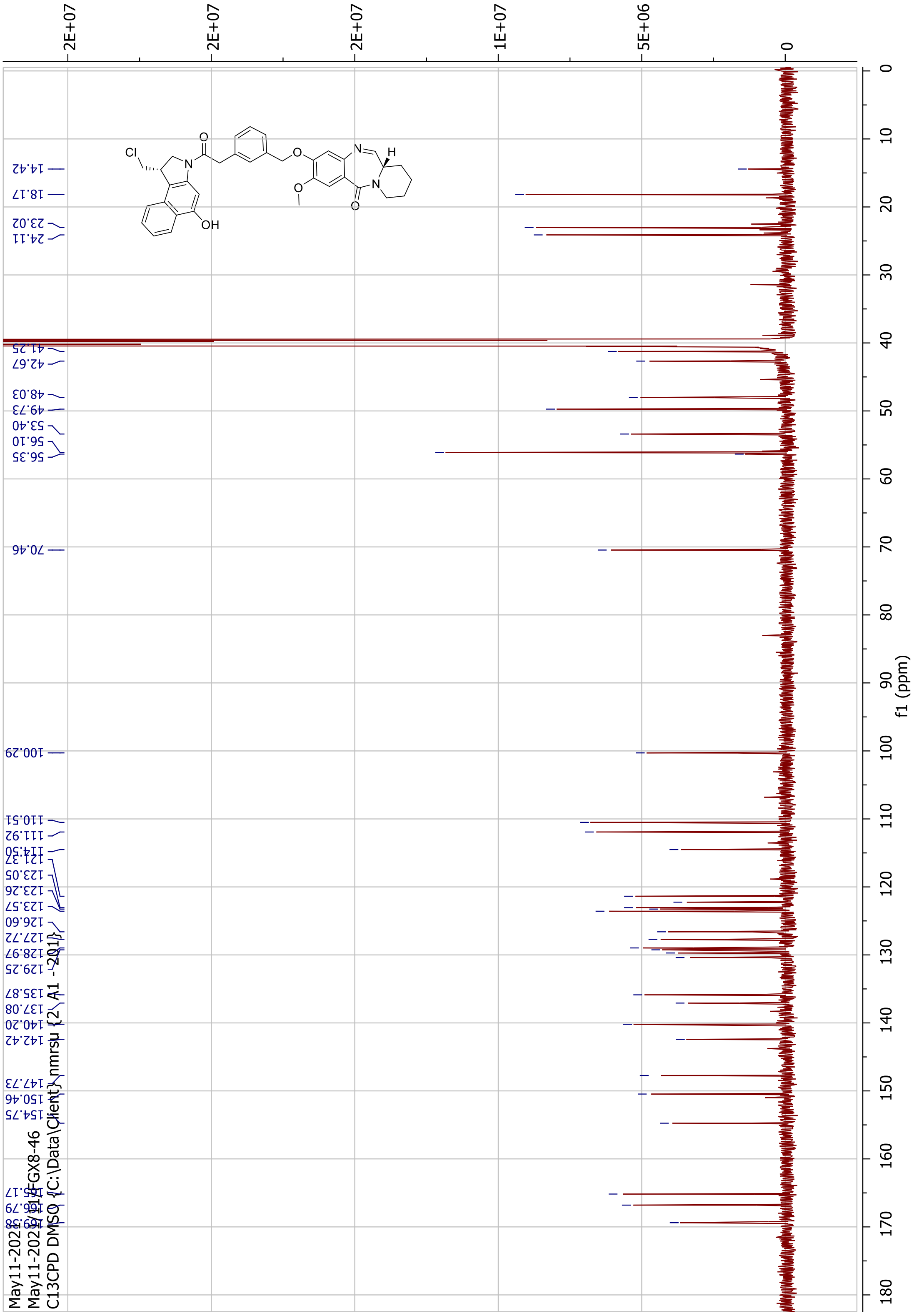

Supplementary Figure S3: LCMS of FGX8-46 (6)

Sample Information Z:\LCMS-XR\2019\201907\20190702\FGX-1.lcd

Sample Name	:	Acquired by	: System Administr
Tray#	: 1	Vial#	: 96
Date Acquired	: 2019/7/3 3:09:48	Injection Volume	: 20
Method File	: LCMS-03-P2-0.5.lcm	Data File	: FGX-1.lcd
Processed by	: System Administrator	Level#	: 0
Report Format File	: D:\REPORT\Report-2020-20180706.lsr		

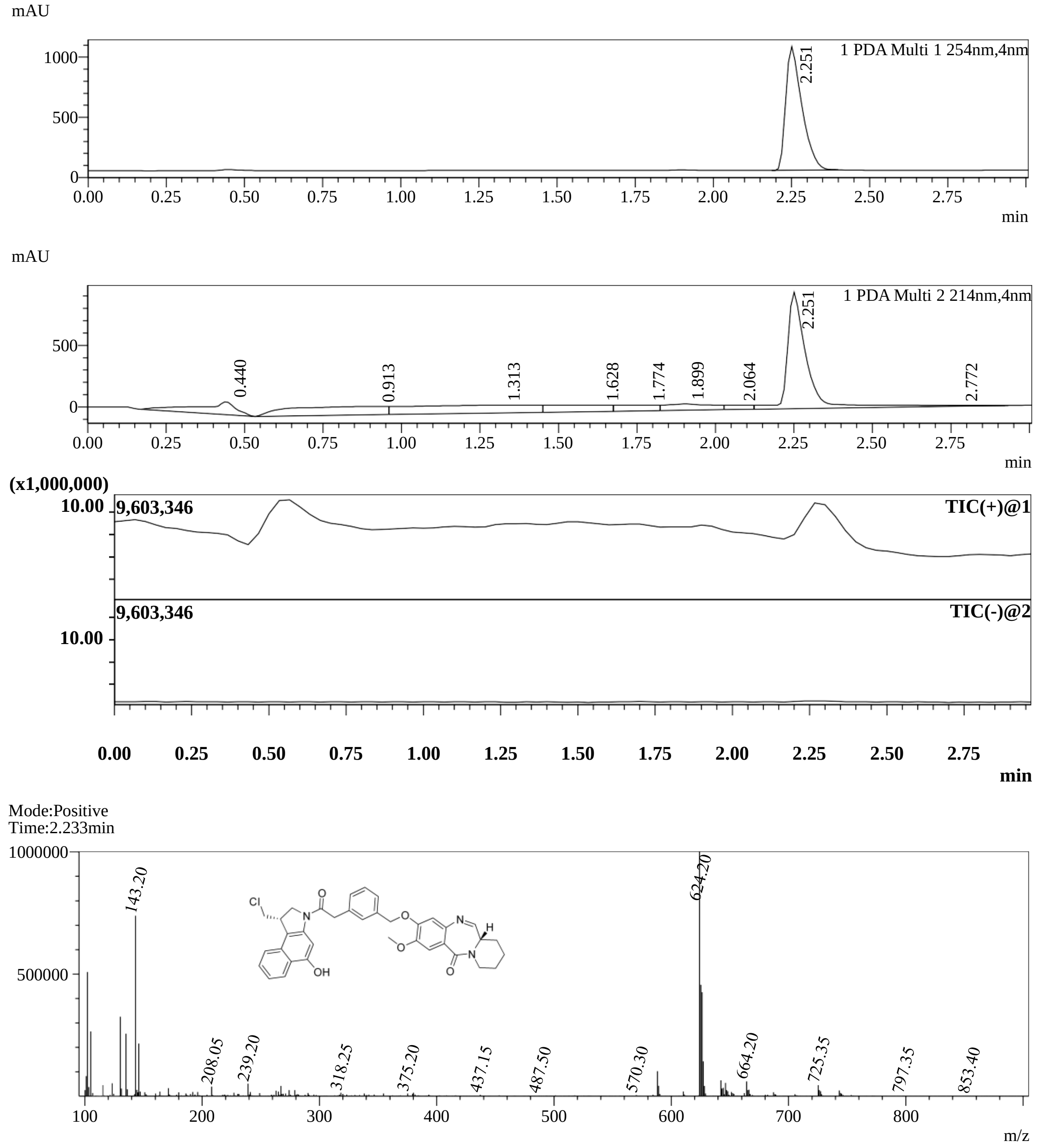

Supplementary Figure S4: Analytical HPLC of FGX8-46 (6)

```
Data File Z:\AGILENT...-1260\2019\201907\20190705\20190705 2019-07-05 09-16-45

=====================================================================
Seq. Line :      1
Acq. Instrument : LC1260                          Location : Vial 45
Injection Date  : 7/5/2019 9:23:23 AM                   Inj :    1
                                                Inj Volume : 2.000 µl
Different Inj Volume from Sequence !      Actual Inj Volume : 10.000 µl
Acq. Method     : D:\DATA\2019\201907\20190705\20190705 2019-07-05 09-16-45\HPLC-01-A2-
                  20160118.M
Last changed    : 7/5/2019 9:16:45 AM
Analysis Method : C:\CHEM32\1\METHODS\DEF_LC.M
Last changed    : 7/5/2019 10:02:07 AM
                  (modified after loading)
```

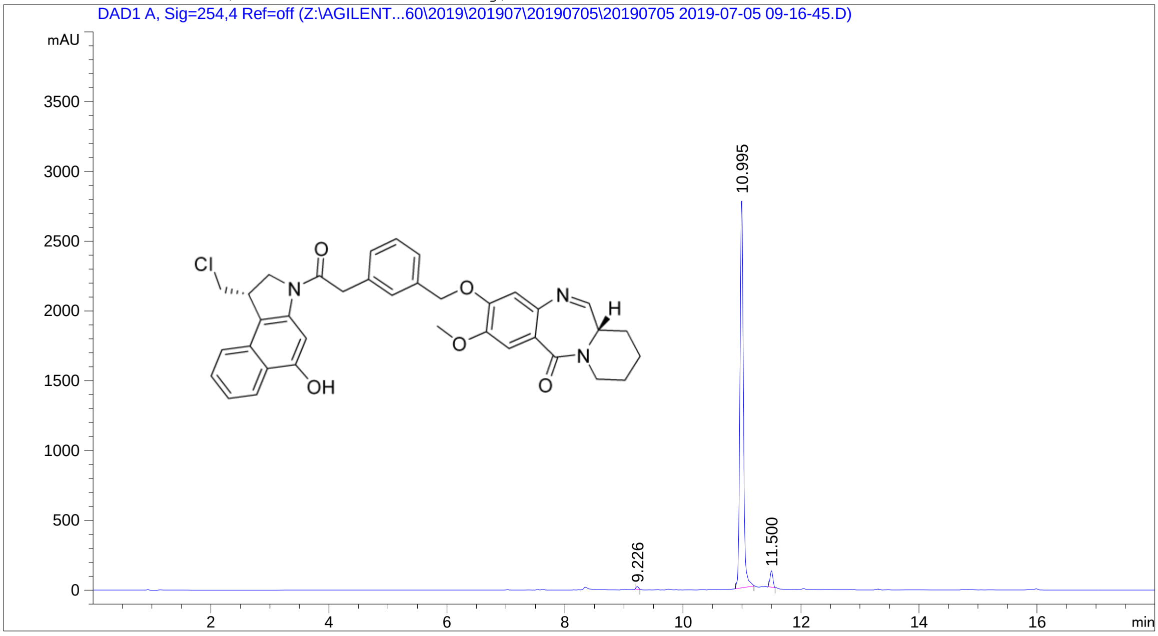


```
=====================================================================
                         Area Percent Report
=====================================================================

Sorted By             :      Signal
Multiplier:                    :      1.0000
Dilution:                      :      1.0000
Use Multiplier & Dilution Factor with ISTDs


Signal 1: DAD1 A, Sig=254,4 Ref=off

Peak RetTime Type  Width     Area      Height     Area
  #    [min]        [min]   [mAU*s]     [mAU]       %
----|-------|----|-------|----------|----------|--------|
   1   9.226 MM R  0.0512   61.34828   19.95389   0.5298
   2  10.995 MM R  0.0664 1.11369e4  2794.52637  96.1709
   3  11.500 MM R  0.0538  382.06970  118.26079   3.2993

Totals :                  1.15804e4  2932.74104


=====================================================================
                          *** End of Report ***
```

Supplementary Figure S5: HRMS of FGX8-46 (6)

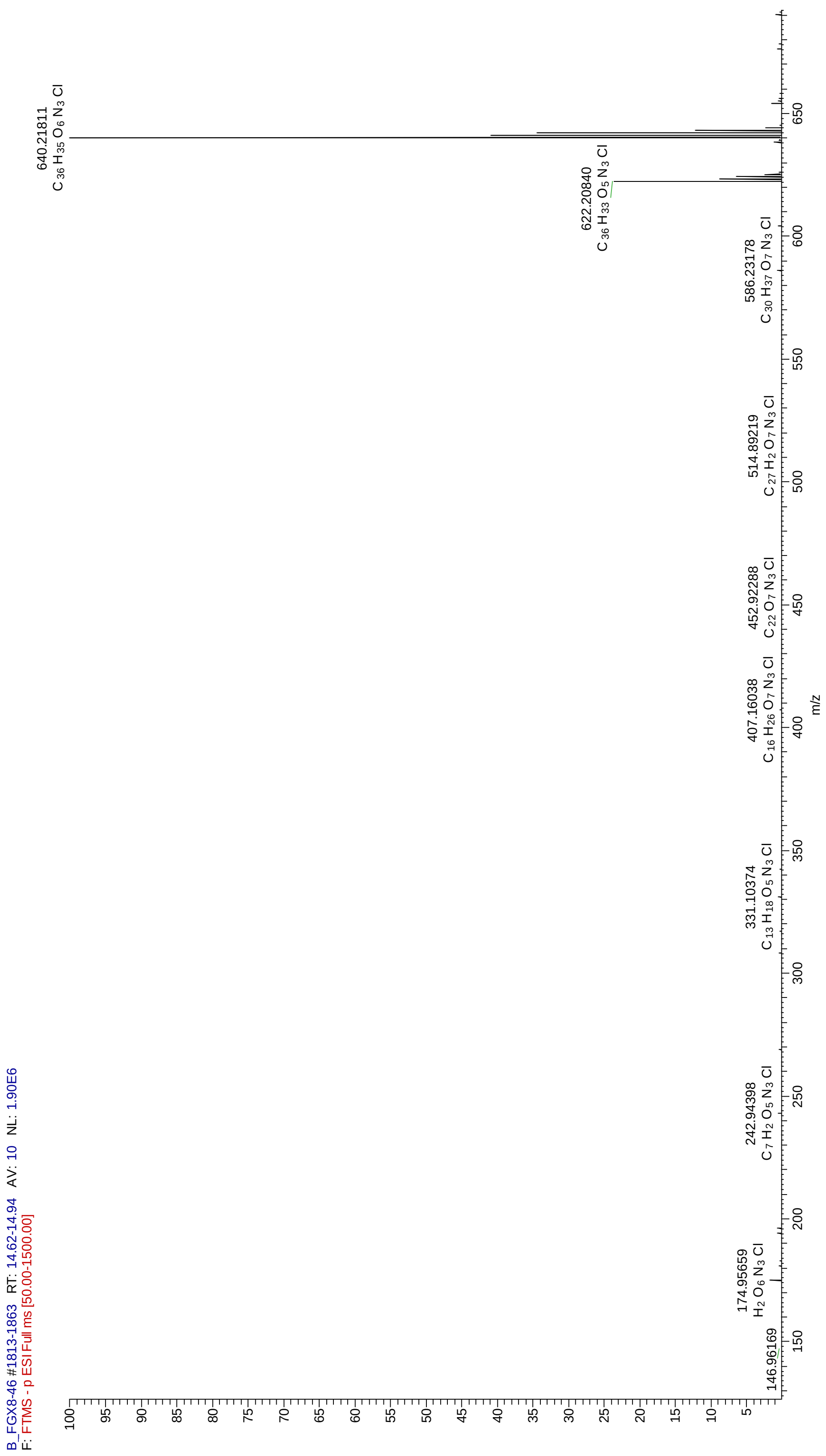

Supplementary Results S6: Characterisation Data for FGX8-46 (6)

(*S*)-3-((3-(2-((*S*)-1-(Chloromethyl)-5-hydroxy-1,2-dihydro-3*H*-benzo[*e*]indol-3-yl)-2-oxoethyl)benzyl)oxy)-2-methoxy-7,8,9,10-tetrahydrobenzo[*e*]pyrido[1,2-*a*][1,4]diazepin-12(6a*H*)-one

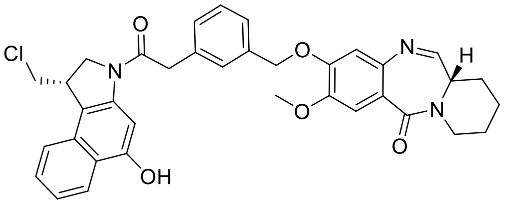

^{1}H NMR (400 MHz, acetone-d_6) δ 9.34 (br s, 1H), 8.21 (d, *J*=8.6 Hz, 1H), 8.10 (br s, 1H), 7.92 (d, *J*=5.9 Hz, 1H), 7.80 (d, *J*=7.8 Hz, 1H), 7.75-7.67 (m, 1H), 7.65-7.59 (m, 1H), 7.57-7.50 (m, 2H), 7.40 (d, *J*=7.0 Hz, 1H), 7.38-7.34 (m, 2H), 6.84 (br, 1H), 5.26-5.12 (m, 1H), 5.13-5.06 (m, 1H), 4.45-4.39 (m, 2H), 4.37-4.31 (m, 2H), 4.14-4.09 (m, 2H), 4.00-3.93 (m, 2H), 3.82 (s, 3H), 3.73-3.70 (s, 1H), 3.67-3.60 (m, 1H), 2.50 (t, *J*=7.4 Hz, 1H), 2.32 (dt, *J*=7.4, 2.0 Hz, 1H), 1.82-1.77 (m, 2H), 1.64-1.55 (br, 2H); ^{13}C NMR (100 MHz, acetone-d_6) δ 169.1, 166.8, 163.9, 150.5, 148.0, 140.2, 135.4, 131.8, 129.1, 128.7, 128.6, 128.5, 126.0, 123.3, 122.9, 122.4, 121.5, 114.6, 111.8, 110.6, 100.3, 70.2, 55.4, 53.2, 49.6, 46.8, 42.7, 41.7, 39.2, 29.7, 24.1, 22.9, 18.1; MS (ES+): *m/z* = 624 $(M+H)^+$; LCMS (**Method A**): t_R = 7.60 min; HRMS calculated for $[C_{36}H_{35}ClN_3O_5]^+$: 624.2260, found: 624.2254.

Supplementary Figure S7A: Partial Transcription Factor (TF) Array Data Set

Some of the key transcription factors modulated in HELA cells, showing a selection of down- and up-regulated TFs. Two key observations are that more TFs are down- rather than up-regulated, and that for the down-regulated TFs, the extent of down-regulation is significantly greater (by up to 12-fold) compared with the up-regulated TFs.

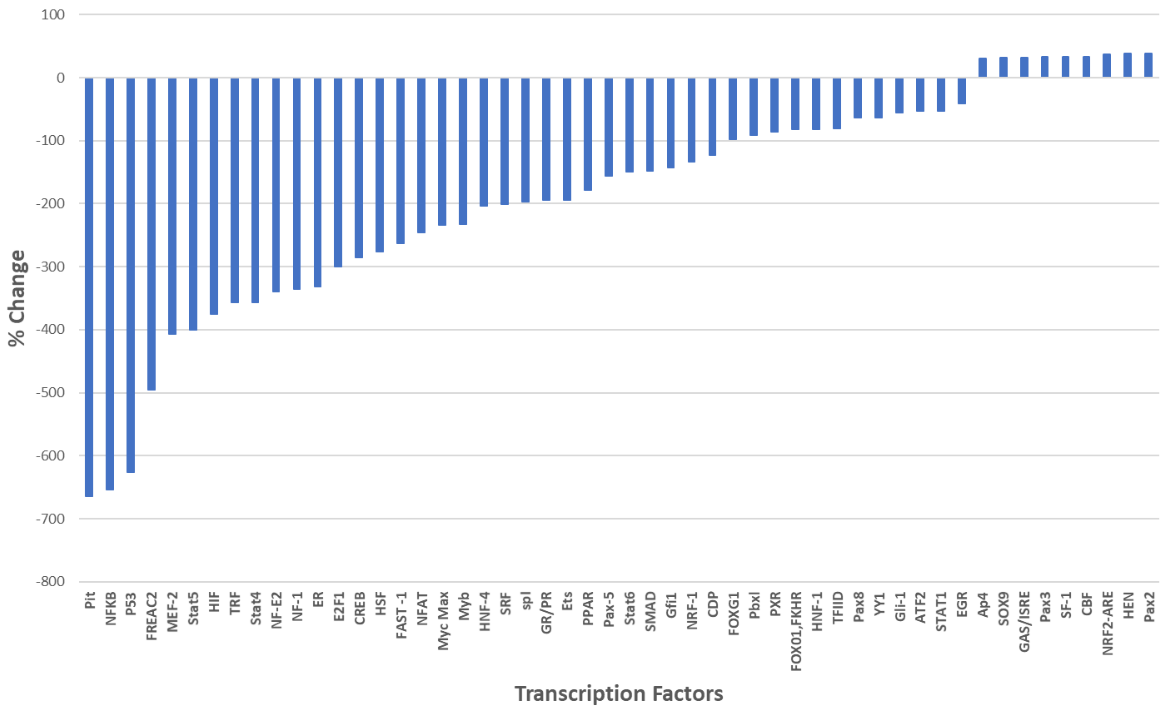

Supplementary Figure S7B: Full Transcription Factor (TF) Array Data Set (95 TFs)

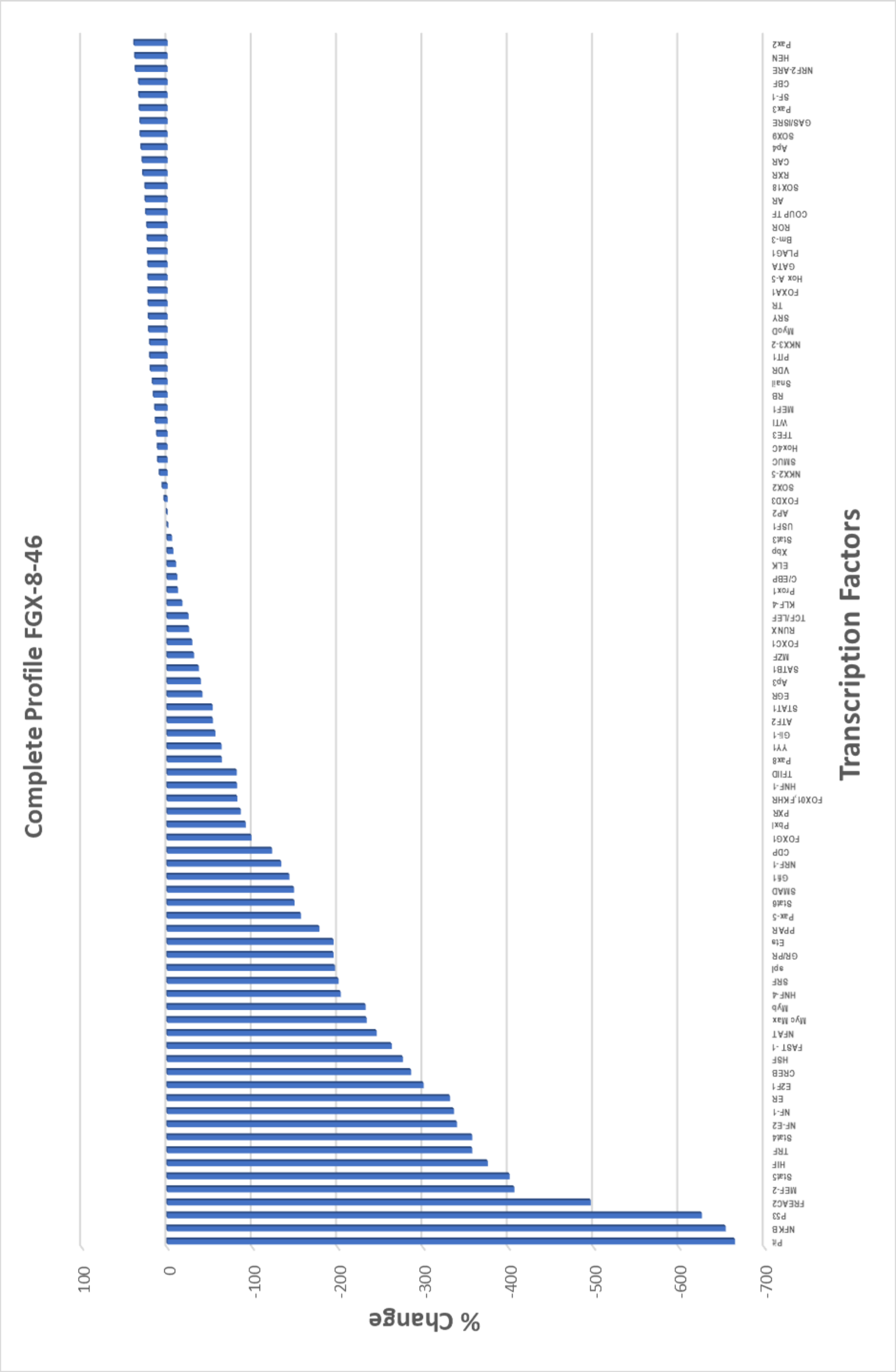

Supplementary Figure S8: ^{1}H NMR of FGX16-11 (9)

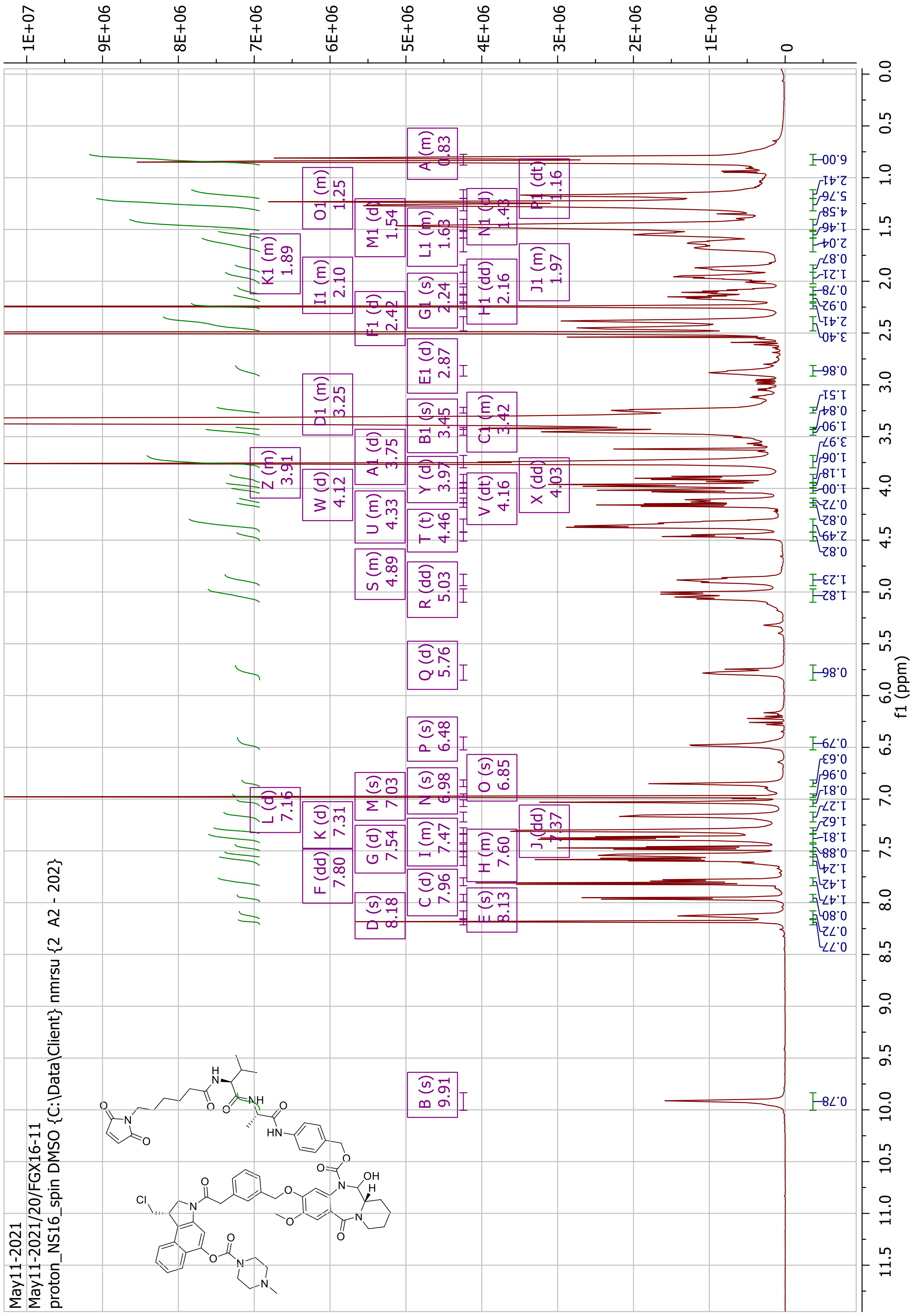

Supplementary Figure S9: ^{13}C NMR of FGX16-11 (9)

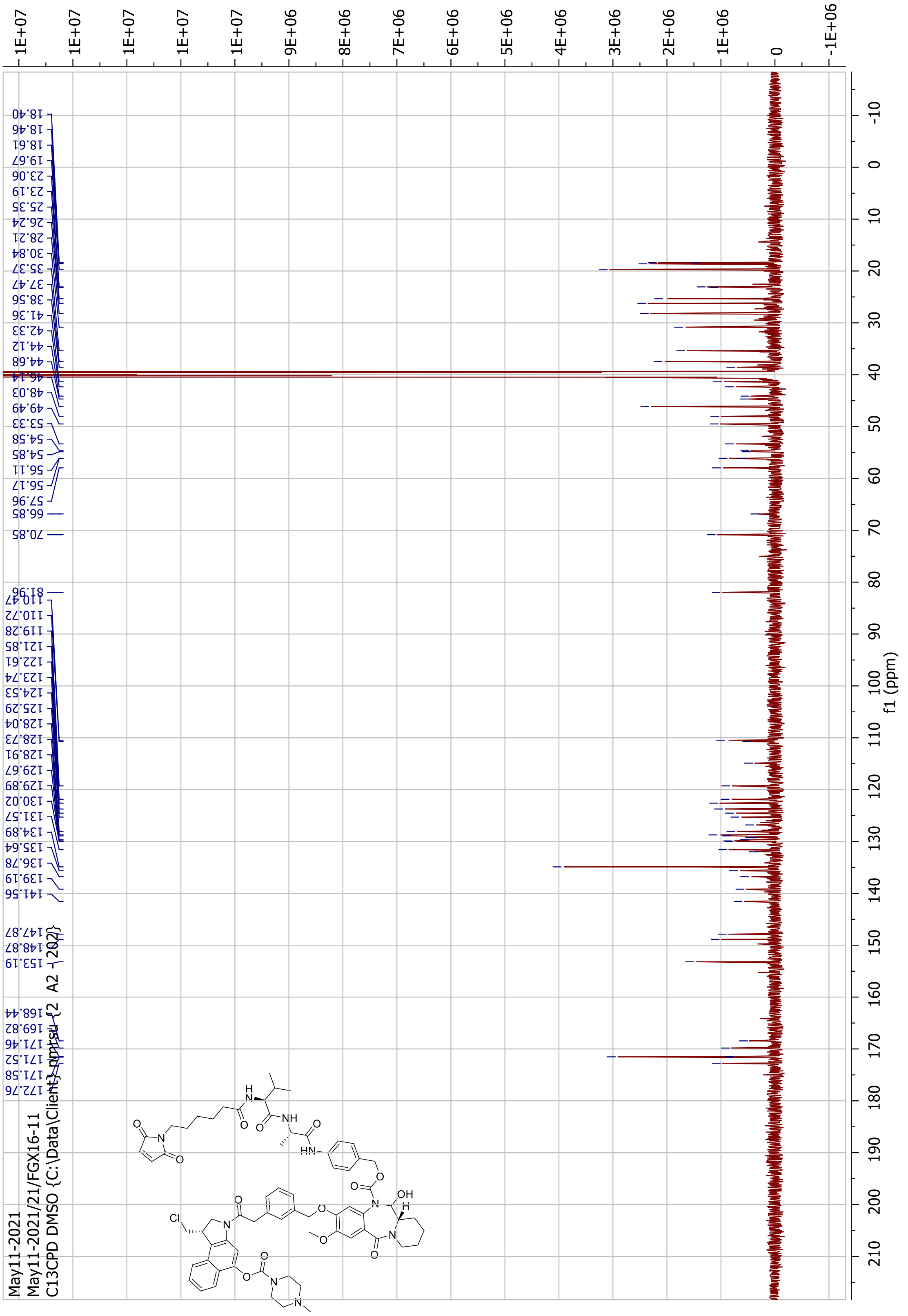

Supplementary Figure S10: LCMS of FGX16-11 (9)

Sample Information Z:\LCMS-2020\2019\201905\20190524.lcd

Acquired by : System Administrator
Tray# : 1 Vial# : 91
Date Acquired : 2019/5/24 9:29:47 Injection Volume
Method File : LCMS-03-P2-POS-M1700.lcm
Processed by : System Administrator Level# 0
Report Format File : D:\LCMS\TEMPLATE\REPORT\Report-2020-20180706.lsr

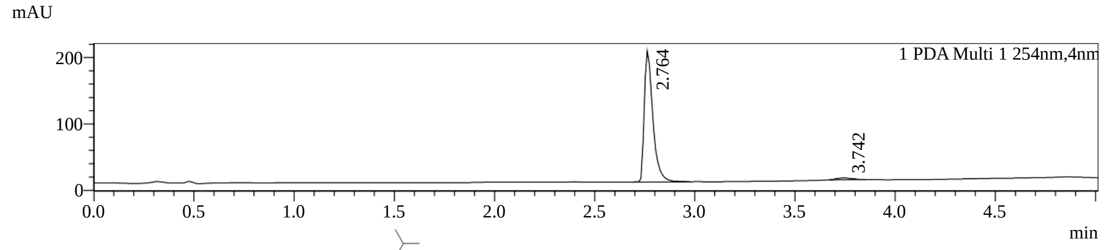

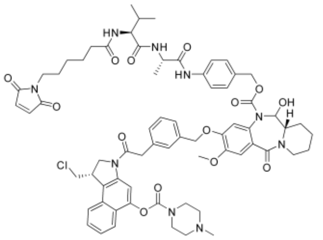

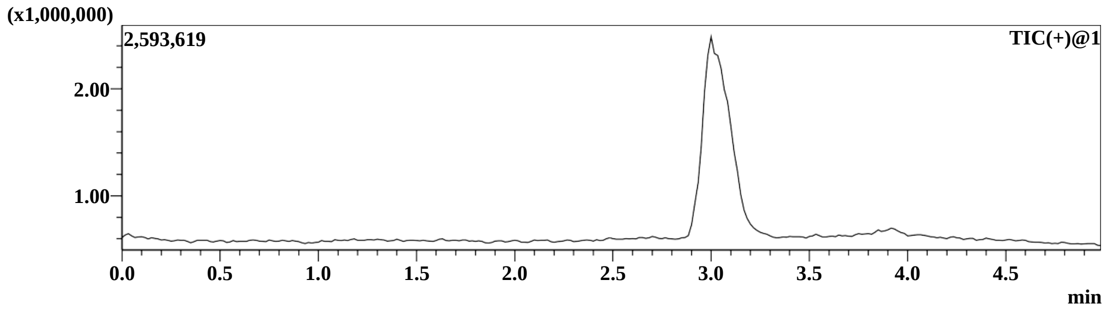

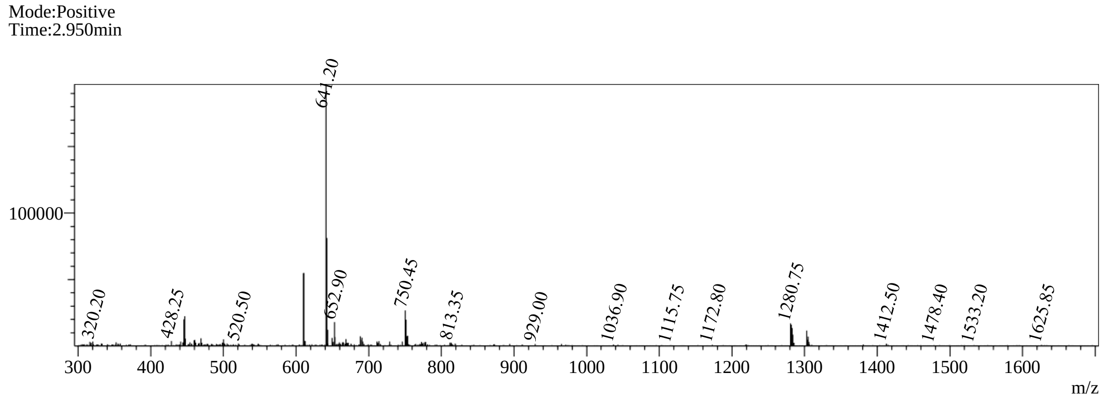

Supplementary Figure S11: Analytical HPLC of FGX16-11 (9)

Data File Z:\AGILENT...-2019\201905\20190524\20190524 2019-05-24 10-30-59.D

```
=====================================================================
Acq. Operator   :                               Seq. Line :   1
Acq. Instrument : Instrument 1                   Location : Vial 92
Injection Date  : 5/24/2019 10:37:04 AM               Inj :   1
                                               Inj Volume : 5.0 µl
Different Inj Volume from Sequence !     Actual Inj Volume : 15.0 µl
Acq. Method     : D:\DATA\201905\20190524\20190524 2019-05-24 10-30-59\HPLC-03-A2-20170209.M
Last changed    : 5/23/2019 9:18:56 AM
Analysis Method : C:\CHEM32\1\METHODS\DEF_LC.M
Last changed    : 5/24/2019 11:37:00 AM
                  (modified after loading)
```

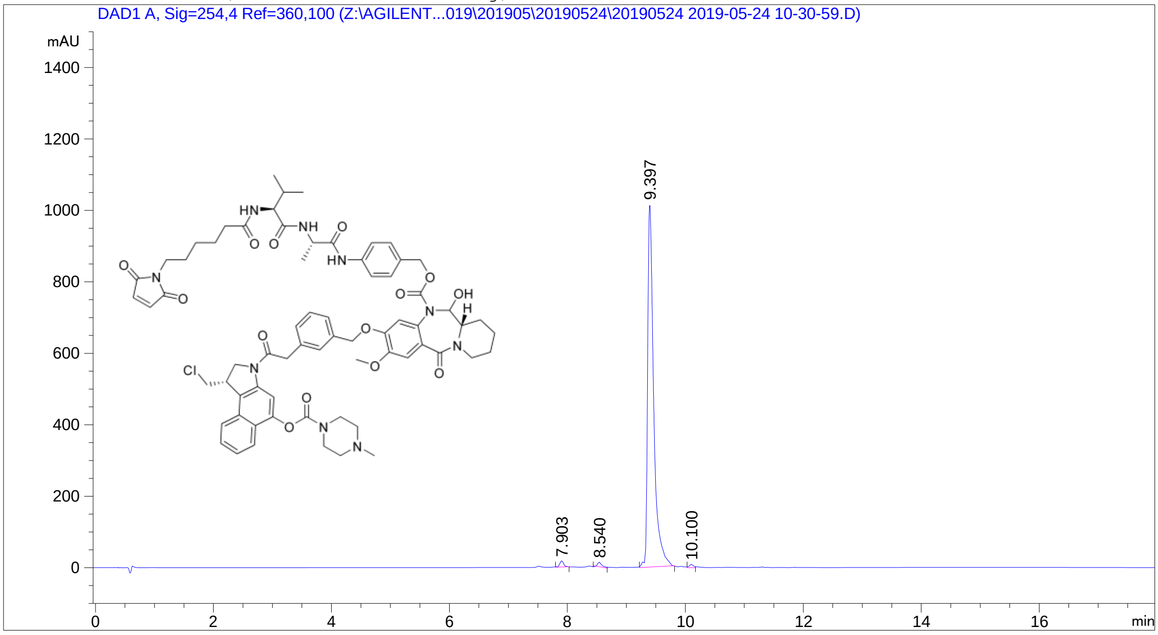


```
=====================================================================
                        Area Percent Report
=====================================================================

Sorted By             :      Signal
Multiplier:                  :      1.0000
Dilution:                    :      1.0000
Use Multiplier & Dilution Factor with ISTDs


Signal 1: DAD1 A, Sig=254,4 Ref=360,100
```

Peak #	RetTime [min]	Type	Width [min]	Area [mAU*s]	Height [mAU]	Area %
1	7.903	MM R	0.0762	77.64870	16.97637	1.0536
2	8.540	MM R	0.0842	63.54946	12.57407	0.8623
3	9.397	MM R	0.1027	7184.75098	1015.27460	97.4889
4	10.100	MM R	0.0786	43.86651	9.30489	0.5952

Totals : 7369.81564 1054.12993

```
=====================================================================
                        *** End of Report ***
```

Supplementary Figure S12: HRMS of FGX16-11 (9)

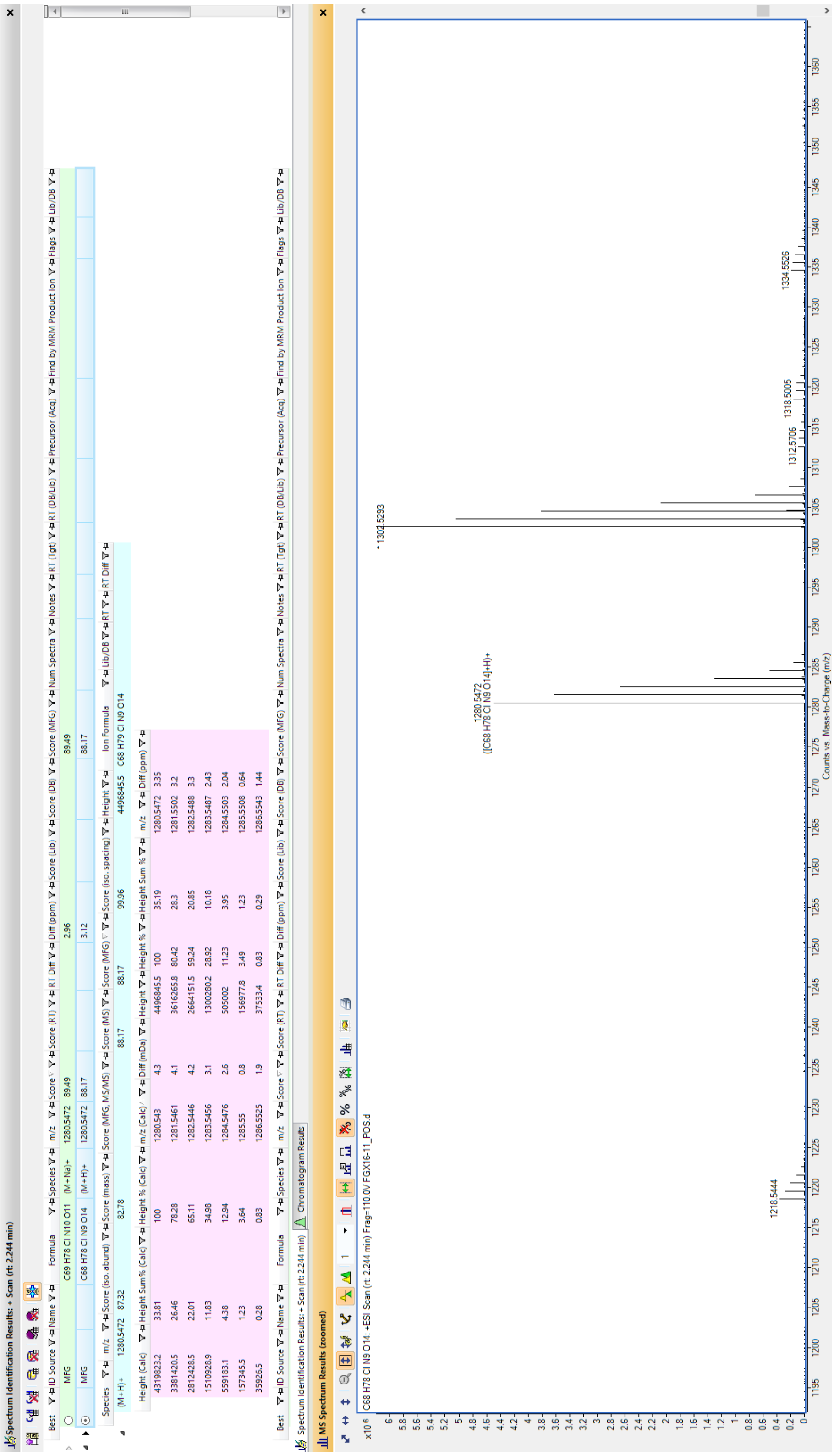

Supplementary Results S13: Characterisation Data for FGX16-11 (9)

4-((*S*)-2-((*S*)-2-(6-(2,5-Dioxo-2,5-dihydro-1*H*-pyrrol-1-yl)hexanamido)-3-methylbutanamido)propanamido)benzyl (6a*S*)-3-((3-(2-((*S*)-1-(chloromethyl)-5-((4-methylpiperazine-1-carbonyl)oxy)-1,2-dihydro-3*H*-benzo[*e*]indol-3-yl)-2-oxoethyl)benzyl)oxy)-6-hydroxy-2-methoxy-12-oxo-6,6a,7,8,9,10-hexahydrobenzo[*e*]pyrido[1,2-*a*][1,4]diazepine-5(12*H*)-carboxylate

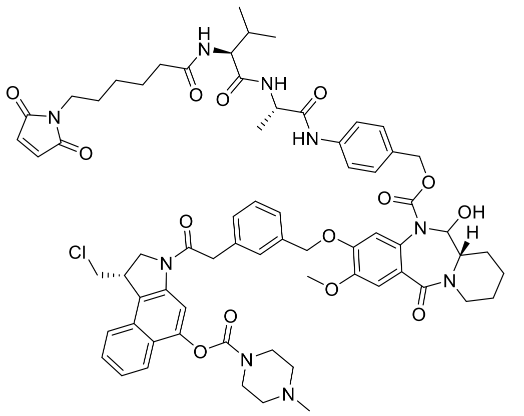

^{1}H NMR (400 MHz, acetone-d_6) δ 9.38 (s, 1H), 8.37 (s, 1H), 7.91 (apparent t, *J*=7.5 Hz, 2H), 7.77 (s, 1H), 7.66 (d, *J*=8.4 Hz, 2H), 7.54 (t, *J*=7.7 Hz, 1H), 7.45-7.37 (m, 3H), 7.30 (br, 4H), 7.22 (d, *J*=7.7 Hz, 2H), 7.07 (s, 1H), 6.80 (s, 2H), 6.73 (s, 1H), 5.96 (d, *J*=10.2 Hz, 1H), 5.24 (d, *J*=11.5 Hz, 1H), 4.89 (d, *J*=12.9 Hz, 1H), 4.74 (d, *J*=12.1 Hz, 1H), 4.47 (br, 3H), 4.30-4.21 (m, 3H), 4.06-3.93 (m, 3H), 3.81 (br, 2H), 3.79 (s, 3H), 3.53 (br, 2H), 3.40 (t, *J*=7.1 Hz, 2H), 3.36 (br, 1H), 2.54-2.36 (m, 4H), 2.26 (s, 3H), 2.08 (s, 4H), 1.77-1.46 (m, 9H), 1.32 (d, *J*=6.4 Hz, 3H), 1.29-1.22 (m, 2H), 1.21-1.17 (m, 1H), 1.06-1.01 (m, 1H), 0.91 (dd, *J*=9.7, 7.0 Hz, 6H); ^{13}C NMR (100 MHz, acetone-d_6) δ 173.5, 171.3, 170.9, 170.8, 168.5, 153.2, 151.2, 149.0, 148.2, 141.4, 139.0, 136.9, 135.1, 134.2, 131.7, 129.9, 129.2, 128.9, 128.6, 127.4, 126.1, 124.6, 123.0, 122.5, 119.2, 115.0, 81.9, 70.5, 66.7, 58.9, 56.0, 55.4, 54.7, 54.4, 53.2, 49.6, 46.9, 45.4, 41.8, 38.3, 37.2, 35.4, 30.4, 29.7, 26.2, 25.1, 23.1, 23.0, 18.8, 17.7; MS (ES+): *m/z* = 1280 (M+H)$^+$; LCMS (**Method A**): t_R = 6.28 min; HRMS calculated for $[C_{68}H_{79}ClN_9O_{14}]^+$: 1280.5430, found: 1280.5472.

Supplementary Figure S14: HIC Chromatogram of Cetuximab-(FGX16-11) (10)

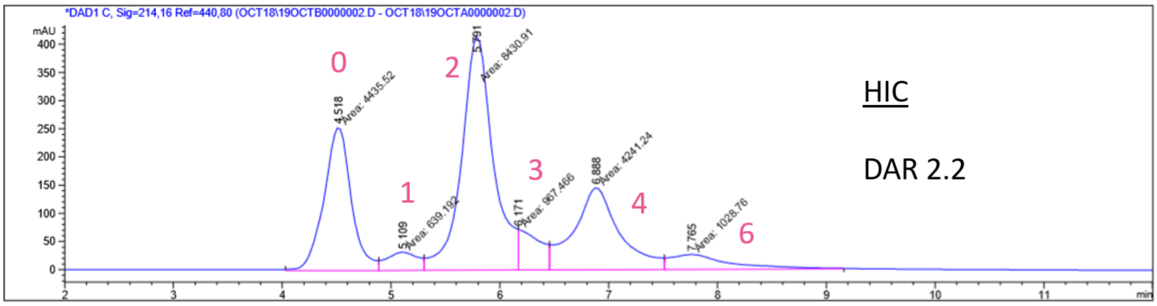

Supplementary Figure S15: SEC chromatogram of Cetuximab-(FGX16-11) (10)

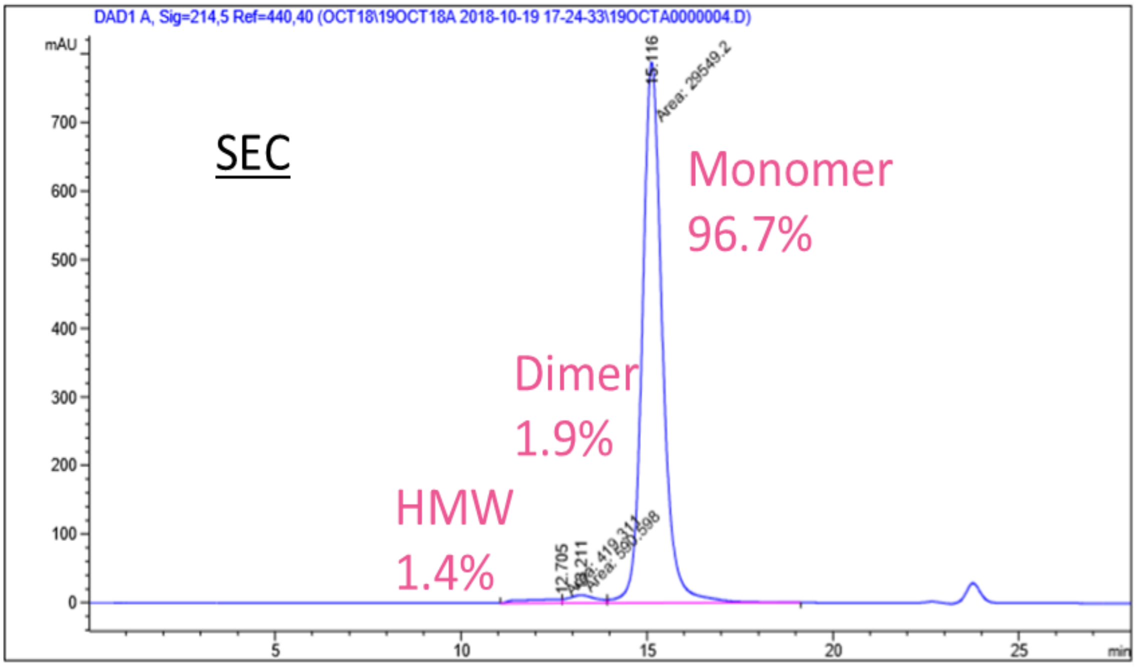

Supplementary Methods S16: Flow Cytometry Gating Strategy

The serial gating strategy employed utilised a primary FSC-A vs SSC-A plot, the exclusion of doublets using FSC-A vs FSC-H and then a bivariate plot showing Annexin V-APC vs 7-AAD. “positive” and “negative” event gating was performed in a pragmatic fashion *i.e.* in all cases there were Annexin V+ and Annexin V- events and 7-AAD+ and 7-AAD- events in the plots. In all cases the gate was placed between the two distinct populations.

Supplementary Figure S17: Gating Strategy

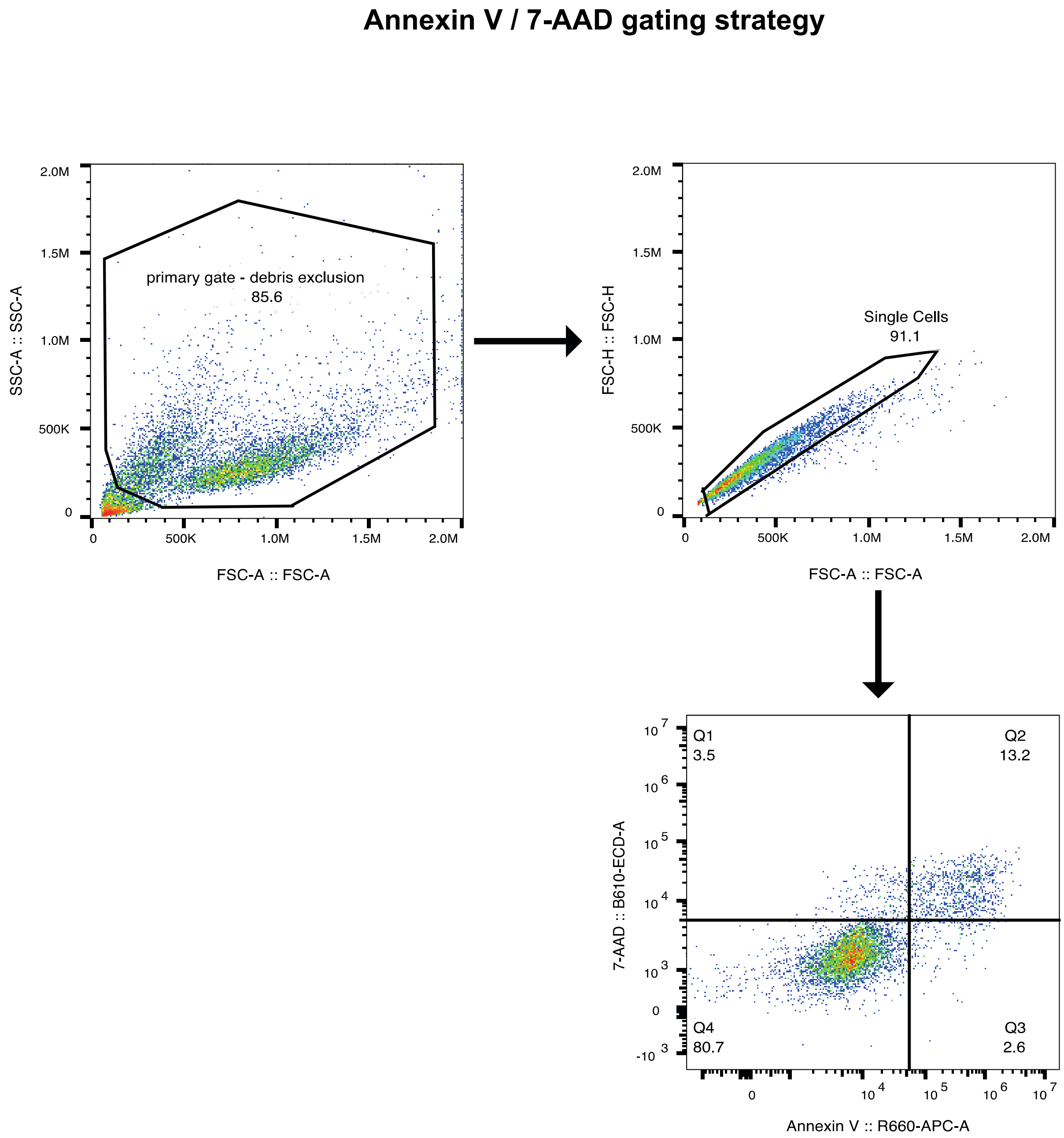

Supplement: Supplementary file 1 — Supplementary Information [file 42003_2022_3633_MOESM1_ESM.pdf]
