# Supplementary material for: DNA sequence-selective G-A cross-linking ADC payloads for use in solid tumour therapies
Source: Commun Biol. 2022 Jul 29;5:741. doi: 10.1038/s42003-022-03633-0 (PMC9338023; doi:10.1038/s42003-022-03633-0)
Supplement: Supplementary file 2 — Description of Additional Supplementary Files [file 42003_2022_3633_MOESM2_ESM.pdf]

Description of Additional Supplementary Files

File name: Supplementary Data 1-4
Description: The source data behind the graphs in the paper.
