# Supplementary material for: DNA sequence-selective G-A cross-linking ADC payloads for use in solid tumour therapies
Source: Commun Biol. 2022 Jul 29;5:741. doi: 10.1038/s42003-022-03633-0 (PMC9338023; doi:10.1038/s42003-022-03633-0)
Supplement: Supplementary file 7 — Reporting Summary [file 42003_2022_3633_MOESM7_ESM.pdf]

nature research

Corresponding author(s): George Procopiou, Paul J. M. Jackson, Daniella di Mascio, Jennifer L. Auer, Chris Pepper, Khondaker Miraz Rahman, Keith R. Fox and David E. Thurston

Last updated by author(s): Mar 13, 2022

Reporting Summary

Nature Research wishes to improve the reproducibility of the work that we publish. This form provides structure for consistency and transparency in reporting. For further information on Nature Research policies, see our Editorial Policies and the Editorial Policy Checklist.

Statistics

For all statistical analyses, confirm that the following items are present in the figure legend, table legend, main text, or Methods section.

n/a	Confirmed	
☐	☒	The exact sample size (*n*) for each experimental group/condition, given as a discrete number and unit of measurement
☐	☒	A statement on whether measurements were taken from distinct samples or whether the same sample was measured repeatedly
☒	☐	The statistical test(s) used AND whether they are one- or two-sided<br>*Only common tests should be described solely by name; describe more complex techniques in the Methods section.*
☒	☐	A description of all covariates tested
☒	☐	A description of any assumptions or corrections, such as tests of normality and adjustment for multiple comparisons
☒	☐	A full description of the statistical parameters including central tendency (e.g. means) or other basic estimates (e.g. regression coefficient) AND variation (e.g. standard deviation) or associated estimates of uncertainty (e.g. confidence intervals)
☒	☐	For null hypothesis testing, the test statistic (e.g. *F*, *t*, *r*) with confidence intervals, effect sizes, degrees of freedom and *P* value noted<br>*Give P values as exact values whenever suitable.*
☒	☐	For Bayesian analysis, information on the choice of priors and Markov chain Monte Carlo settings
☒	☐	For hierarchical and complex designs, identification of the appropriate level for tests and full reporting of outcomes
☒	☐	Estimates of effect sizes (e.g. Cohen's *d*, Pearson's *r*), indicating how they were calculated

Our web collection on statistics for biologists contains articles on many of the points above.

Software and code

Policy information about availability of computer code

Data collection	N/A
Data analysis	N/A

For manuscripts utilizing custom algorithms or software that are central to the research but not yet described in published literature, software must be made available to editors and reviewers. We strongly encourage code deposition in a community repository (e.g. GitHub). See the Nature Research guidelines for submitting code & software for further information.

Data

Policy information about availability of data

All manuscripts must include a data availability statement. This statement should provide the following information, where applicable:

- Accession codes, unique identifiers, or web links for publicly available datasets
- A list of figures that have associated raw data
- A description of any restrictions on data availability

There are no restrictions on the availability of the raw data. Interested parties can e-mail the corresponding author directly.

Field-specific reporting

Please select the one below that is the best fit for your research. If you are not sure, read the appropriate sections before making your selection.

☒ Life sciences ☐ Behavioural & social sciences ☐ Ecological, evolutionary & environmental sciences

For a reference copy of the document with all sections, see nature.com/documents/nr-reporting-summary-flat.pdf

Life sciences study design

All studies must disclose on these points even when the disclosure is negative.

Sample size	N/A
Data exclusions	N/A
Replication	Where relevant, experiments were repeated twice (i.e., each experiment carried out a total of three times).
Randomization	N/A
Blinding	N/A

Reporting for specific materials, systems and methods

We require information from authors about some types of materials, experimental systems and methods used in many studies. Here, indicate whether each material, system or method listed is relevant to your study. If you are not sure if a list item applies to your research, read the appropriate section before selecting a response.

Materials & experimental systems

n/a	Involved in the study	
☐	☒	Antibodies
☐	☒	Eukaryotic cell lines
☒	☐	Palaeontology and archaeology
☐	☒	Animals and other organisms
☒	☐	Human research participants
☒	☐	Clinical data
☐	☒	Dual use research of concern

Methods

n/a	Involved in the study	
☒	☐	ChIP-seq
☐	☒	Flow cytometry
☒	☐	MRI-based neuroimaging

Antibodies

Antibodies used	Cetuximab
Validation	The antibody was commercially obtained with quality control data provided by the vendor.

Eukaryotic cell lines

Policy information about cell lines

Cell line source(s)	All human cell lines from ATCC (by IRMB CRO). SK-BR-3 ATCC, MCF7 ATCC, ZR 75-1 ATCC, U138-MG ATCC, Reh ATCC, SW48 ATCC, SW620 ATCC, LIM1215 ATCC, Raji ATCC, JVM2 ATCC, Jurkat ATCC
Authentication	None of the cell-lines were authenticated
Mycoplasma contamination	All tested positive for mycoplasma
Commonly misidentified lines (See ICLAC register)	

The cell line was originally thought to be a derivative of MCF-7. When found to come from a different donor, the cell line was re-named NCI/ADR-RES (Scudiero et al, 1998; PMID 9625176). SNP analysis has subsequently shown that the cell line is genetically identical to OVCAR-8.
The cell line was originally thought to be a derivative of MCF-7. When found to come from a different donor, the cell line was re-named NCI/ADR-RES (Scudiero et al, 1998; PMID 9625176). SNP analysis has subsequently shown that the cell line is genetically identical to OVCAR-8.

Capes-Davis et al compared STR profiles to show that YMB-1 and ZR-75-1 came from the same donor. ZR-75-1 was established from a 63 year old female and first published in 1978. YMB-1 was established by a different laboratory from a 55 year old female and was first published in 1984. Based on the different establishment locations and dates, it is likely that YMB-1 is misidentified. [Source: ATCC CRL-1500; JCRB0823; Engel et al, 1978, PMID 688225; Yamane et al, 1984, PMID 6534930; Capes-Davis et al, 2013.]

Boonstra et al. refer to the contaminant as SW620. However, SW-480 and SW-620 were derived from the same individual, so both carry the same identity; the contaminating cell line could be either of these two cell lines.

Website states, "The NC-37 was originally established as a unique cell line from caucasian male. But later it was found to be cross-contaminated by the Raji cell reported by the ATCC. However we decided to keep the NC-37 on our list of cell lines because it have been used under the name by many experiments in the world."

Reviewed by ICLAC (ref: 171204). Samples of the five cell lines in the corrigendum were supplied to ICLAC for STR profiling by Dr Michael Kent (UCSD) and tested by Erin Hall (Genetica DNA laboratories). STR profiles for UCDK9B1, UCDK9B2, UCDK9B3, UCDK9B4, and UCDK9B5 demonstrated that Jurkat is the parental cell line in each case.

Animals and other organisms

Policy information about studies involving animals; ARRIVE guidelines recommended for reporting animal research

Laboratory animals	ICR-CD1 Mice
Wild animals	N/A
Field-collected samples	N/A
Ethics oversight	N/A

Note that full information on the approval of the study protocol must also be provided in the manuscript.

Dual use research of concern

Policy information about dual use research of concern

Hazards

Could the accidental, deliberate or reckless misuse of agents or technologies generated in the work, or the application of information presented in the manuscript, pose a threat to:

No	Yes	
☒	☐	Public health
☒	☐	National security
☒	☐	Crops and/or livestock
☒	☐	Ecosystems
☒	☐	Any other significant area

Experiments of concern

Does the work involve any of these experiments of concern:

No	Yes	
☒	☐	Demonstrate how to render a vaccine ineffective
☒	☐	Confer resistance to therapeutically useful antibiotics or antiviral agents
☒	☐	Enhance the virulence of a pathogen or render a nonpathogen virulent
☒	☐	Increase transmissibility of a pathogen
☒	☐	Alter the host range of a pathogen
☒	☐	Enable evasion of diagnostic/detection modalities
☒	☐	Enable the weaponization of a biological agent or toxin
☒	☐	Any other potentially harmful combination of experiments and agents

Flow Cytometry

Plots

Confirm that:

☒ The axis labels state the marker and fluorochrome used (e.g. CD4-FITC).

☒ The axis scales are clearly visible. Include numbers along axes only for bottom left plot of group (a 'group' is an analysis of identical markers).

☒ All plots are contour plots with outliers or pseudocolor plots.

☒ A numerical value for number of cells or percentage (with statistics) is provided.

Methodology

Sample preparation	MCF-7 and MDA-MB-231 cells were harvested, washed in phosphate-buffered saline and resuspended in binding buffer and containing 4µL of annexin V labelled with allophycocyanin (APC) (Biolegend). Cells were subsequently labelled with 7-AAD and apoptosis was quantified using a CytoFLEX LX flow cytometer (Beckman Coulter). At least 10,000 events were acquired and data were subsequently analysed using FlowJo software. LD50 values (the concentration of FGX8-46 required to kill 50% of cells) were interpolated from the dose-response curves.
Instrument	Cellular events were acquired on a CytoFLEX LX flow cytometer (Beckman Coulter).
Software	The dedicated flow cytometry software installed on the CytoFLEX LX is CytExpert version 2.4.0.28. Subsequently, FCS files were exported and further analysed using FlowJo v.10 software.
Cell population abundance	No flow sorting was performed - only benchtop flow cytometric analysis. The abundance of the cell types under investigation were effectively 100% as the cells analysed were monoclonal cell lines. The abundance of apoptotic cell death measured in each analyte was dependent on the concentration of agent addded to each of the cultures.
Gating strategy	The serial gating strategy employed utilised a primary FSC-A vs SSC-A plot, the exclusion of doublets using FSC-A vs FSC-H and then a bivariate plot showing Annexin V-APC vs 7-AAD. "positive" and "negative" event gating was performed in a pragmatic fashion i.e. in all cases there were Annexin V+ and Annexin V- events and 7-AAD+ and 7-AAD- events in the plots. In all cases the gate was placed between the two distinct populations.

☒ Tick this box to confirm that a figure exemplifying the gating strategy is provided in the Supplementary Information.
